# LOW DOSE OF ESMOLOL ATTENUATES SEPSIS-INDUCED IMMUNOSUPPRESSION *VIA* MODULATING T-LYMPHOCYTE APOPTOSIS AND DIFFERENTIATION

**DOI:** 10.1097/SHK.0000000000002104

**Published:** 2023-02-28

**Authors:** Ying Ma, Zhenshun Cheng, Yong Zheng, Wei Wang, Shaojun He, Xiaolian Zhou, Jiong Yang, Chaojie Wei

**Affiliations:** ∗ Department of Pulmonary and Critical Care Medicine, Zhongnan Hospital of Wuhan University, Wuhan, China; † Wuhan Research Center for Infectious Diseases and Cancer, Chinese Academy of Medical Sciences, Wuhan, China; ‡ Hubei Engineering Center for Infectious Disease Prevention, Control and Treatment, Wuhan, China; § Department of Anatomy and Embryology, Wuhan University Taikang Medical School (School of Basic Medical Sciences), Wuhan, China

**Keywords:** Septic shock, esmolol, T-lymphocyte, apoptosis, differentiation

## Abstract

**Background:** Immunosuppression caused by immune cell apoptosis and an imbalance of T helper 2 cells (T_H_2) and T helper 1 cells (T_H_1), is associated with poor outcomes in septic patients. Esmolol was reported to improve survival by modulating immune responses in septic shock. Whether esmolol could alleviate sepsis-induced immunosuppression and the optimal dose are unclear. **Methods:** Four hours after cecal ligation and puncture (CLP), Wistar rats were randomized into CLP, CLP + E-5 (esmolol: 5 mg·kg^−1^·h^−1^) and CLP + E-18 (esmolol: 18 mg·kg^−1^·h^−1^) groups. Eight rats were underwent sham operation. Eighteen hours after CLP, hemodynamics and organ histological injuries were evaluated, peripheral blood mononuclear cells apoptosis and T-lymphocyte subsets counts were determined by flow cytometry, and the expression of p-Akt, Bcl-2, cleaved Caspase-3, and p-Erk1/2 in splenic CD4^+^ T-lymphocytes was determined by western blot and immunohistochemistry. β_1_-Adrenoreceptor expressions were evaluated using real-time polymerase chain reaction and immunohistochemistry. **Results:** Cecal ligation and puncture induced tachycardia, hypotension, hyperlactatemia, and multiple organ injury. Heart rate was unchanged in the CLP + E-5 group but decreased in the CLP + E-18 group. Hypotension, lactatemia, and multiple organ injuries were improved only in the CLP + E-5 group. T-lymphocyte apoptosis and T_H_2/T_H_1 ratio was decreased in CLP + E-5 but not in CLP + E-18. p-Akt and Bcl-2 expressions were increased, while cleaved Caspase-3 and p-Erk1/2 expressions were decreased in CLP + E-5. β_1_-Adrenoreceptor expressions were unchanged in both CLP + E-5 and CLP + E-18 groups. **Conclusions:** Low dose of esmolol reduced T-lymphocyte apoptosis and restored T_H_2/T_H_1 ratio in septic shock. Esmolol might modulate Akt/Bcl-2/Caspase-3 pathway to relieve T-lymphocyte apoptosis and inhibit Erk1/2 activity to decrease T_H_0 differentiation to T_H_2. Esmolol may be a potential immunoregulator of septic shock.

## INTRODUCTION

Septic shock develops as a dysregulated host inflammatory response to infection, resulting in multiple organ dysfunction that is associated with high mortality worldwide (1). Both pro- and anti-inflammatory immune responses occur after the onset of sepsis. If sepsis persists, patients will enter a markedly immunosuppressive state (2). Immunosuppressed septic patients are at a high risk of secondary nosocomial infection resulting in an increasing 13% mortality of septic patients (3). Immune cell apoptosis, such as circulating monocytes, B-lymphocytes and T-lymphocytes, and upregulated T helper 2 cells (T_H_2)/T helper 1 cells (T_H_1) ratio are the most reported characteristics contributing to immunosuppression during septic shock (4,5).

In experimental and clinical studies, esmolol, a highly selective ultrashort-acting β_1_-adrenoreceptor blocker, was recently reported to improve cardiovascular function and survival in septic shock (6–8). The beneficial effects of esmolol in septic shock have been previously considered because of its hemodynamic effects (9). However, recent evidence has shown that the beneficial effects of esmolol in septic shock are associated with immunomodulation (10,11). However, how esmolol influences the immune response in septic shock and the optimal dose are unclear.

**Fig. 1:**
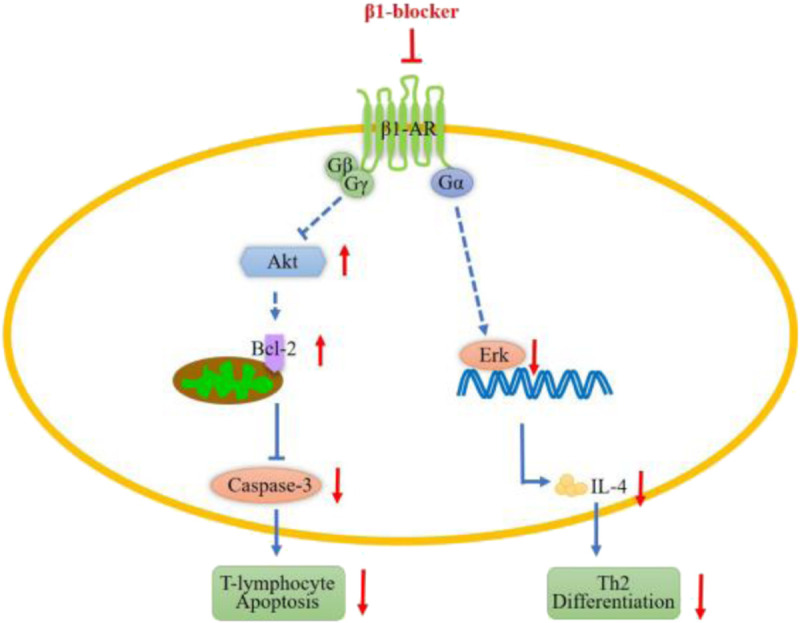
**Signaling pathways of** **β_1_-adrenoreceptors involved in inflammation.** Agonist binding stimulates β_1_-adrenoreceptors and results in coupling with and activation of G protein, which dissociates into Gα and Gβγ subunits. The Gβγ inhibits Akt phosphorylation, which subsequently decrease Bcl-2 release resulting in Caspase-3 cleaved, finally promotes cell apoptosis. The Gα *via* several downstream signaling factors induces activation of Erks transcription factors, which induce IL-4 production promoting T_H_2 differentiation. The red arrows show the effects of blocking β_1_-AR by esmolol on its downstream signaling pathway.

Lymphocytes and monocytes expressed β_1_-adrenoreceptors (12), which belong to the G-protein–coupled receptor (GPCR) super family. The GPCRs couple to a G protein heterotrimer including the α, β, and γ subunits in the intracellular region (Fig. 1). G protein α (Gα) binds to the G protein βγ dimer (Gβγ) in the inactive state. The GPCR activation leads to the dissociation of Gα and Gβγ. Gβγ modulates protein kinase B (Akt) *via* phosphatidylinositide 3-kinases (PI3K) (13). Akt activation leads to B-cell leukemia/lymphoma 2–associated death promoter (Bad) phosphorylation, resulting in B-cell leukemia/lymphoma 2 (Bcl-2) release, ultimately promoting cell survival (14). Our previous study showed that blocking the β_1_-adrenoreceptor by esmolol could increase Akt phosphorylation in cardiovascular tissue (10). Qi et al. reported that Akt phosphorylation decreased sepsis-induced cardiomyocyte apoptosis *via* upregulation of Bcl-2 and downregulation of cleaved Caspase-3 both *in vitro* and *in vivo* (15). Thus, we aimed to determine whether esmolol could reduce sepsis-induced peripheral blood mononuclear cell (PBMC) apoptosis by modulating Akt/Bcl-2/Caspase-3 pathway. Activated Gα induces extracellular regulated protein kinases (Erk) phosphorylation through the adenylyl cyclase–cyclic adenosine monophosphate–protein kinase A pathway (16). Furthermore, Erk is an obligatory mediator of the T_H_2 differentiation pathway (17). Interleukin 4, endogenously produced upon T-cell receptor (TCR) cross-linking, establishes a positive feedback loop through IL-4R that further reinforces IL-4 expression in T_H_0 (18,19), inducing T_H_0 differentiation to T_H_2. Erk promotes T_H_2 differentiation by activating the early phase of TCR-dependent IL-4 production (17). Hence, we also aimed to determine whether esmolol could reduce T_H_0 differentiation to T_H_2 by inhibiting Erk1/2 activation.

## METHODS

### Animals

Adult male Wistar rats weighing 300–400 g were obtained from the Center for Animal Experiments of Wuhan University. All animal experiments were approved by the Institutional Animal Care and Use Committee of the Animal Experiment Center of Wuhan University (E2020072901) and followed the institutional and national guidelines.

### Study design

The cecal ligation and puncture (CLP) model was used to develop a septic shock model as previously described (20). Four hours after CLP, all rats were randomized into three groups (CLP [n = 8], CLP + E-5 [CLP with esmolol infused at 5 mg·kg^−1^·h^−1^, n = 8], CLP + E-18 [CLP with esmolol infused at 18 mg·kg^−1^·h^−1^, n = 8]). Eight rats were under sham operation. Four hours after surgery, all rats received fluid resuscitation (Saline 10 mL·kg^−1^·h^−1^), antibiotic (meropenem 10 mg·kg^−1^), and analgesic (nalbuphine 0.2 mg·kg^−1^·h^−1^) for 14 hours. The rats in the CLP + E-5 and CLP + E-18 groups received the infusion of esmolol initiated 4 hours after surgery for a period of 14 hours. Assessments were performed 18 hours after the CLP or sham surgery. Eighteen-hour mortality was collected in each group.

### Dose selection of esmolol

A high dose with heart rate reduction and a low dose without heart rate reduction were chosen in the study to determine the optimal dose. Infusing septic shock rats with esmolol at 18 kg^−1^·h^−1^ induced heart rate reduction compared with CLP group in the previous study (10). To compare with the previous study, 18 mg·kg^−1^·h^−1^ was chosen as the high dose with heart rate reduction in the study. The low dose was chosen according to our previous study (10). In this study, we did a dose gradient analysis (esmolol infused at 1, 5, and 18 mg·kg^−1^·h^−1^) and found that esmolol infused at both 5 and 1 mg·kg^−1^·h^−1^ did not reduce heart rate but 5 mg·kg^−1^·h^−1^ presented better immunomodulatory effects compared with 1 mg·kg^−1^·h^−1^. Thus, esmolol infused at 5 mg·kg^−1^·h^−1^ was chosen as the low dose without heart rate reduction.

### Hemodynamics and organ injuries measurement

The pressure transducer catheter was inserted into the right carotid artery of anesthetized rats and connected to a BL-420N biological signal recorder (Taimeng, Chengdu, China) to measure the HR and MAP. Arterial blood was collected for lactate detection using an ABL800 FLEX blood-gas Analyzer (Radiometer, Denmark). Heart, lung, and spleen tissue sections were stained with hematoxylin and eosin, and morphological changes were observed under the optical microscope (Olympus, Tokyo, Japan) at 200× magnification. Histopathological lesions were quantified using five randomly selected fields per slide (21).

### Flow cytometry

Peripheral blood mononuclear cells were isolated from all rats using Ficoll-Paque (Cytiva, Sweden). Cell surface markers were used to identify circulating monocytes (CD11a^+^CD11b^+^), B-lymphocytes (CD45RA^+^), T-lymphocytes (CD3^+^), and CD4^+^ T-lymphocytes (CD3^+^CD4^+^) in the PBMCs. According to the manufacturer’s protocol, apoptosis detection was performed using a FITC Annexin V Apoptosis Detection Kit with 7-amino-actinomycin D. After stimulation with the leukocyte activation cocktail, CD4^+^ T-lymphocytes were stained with INF-γ and IL-4 to identify T_H_1 and T_H_2 counts, respectively. Antibody and gating information were shown in Supplemental Table S1 and Supplemental Figure S1 and S2, http://links.lww.com/SHK/B646.

### Western blot

Because of the limited numbers of circulating T-lymphocytes, splenic CD4^+^ T-lymphocytes were used. Splenic CD4^+^ T-lymphocytes were purified using Rat CD4 microbeads (Miltenyi Biotec, Bergisch Gladbach, Germany) following the manufacturer’s instructions. The purity of splenic CD4^+^ T-lymphocytes was determined by flow cytometry (Beckman Coulter, Indianapolis IN) using PE/CY7-labeled anti-CD4 antibody (Supplemental Table S1 and Supplemental Fig. S3, http://links.lww.com/SHK/B646).

Protein was extracted from isolated splenic CD4^+^ T-lymphocytes from all rats. After separation on an SDS-PAGE gel, the protein samples were transferred to a PVDF membrane (Millipore) and incubated with the following antibodies: anti-phosphorylated-Akt (p-Akt), anti-Akt, anti-phosphorylated-Erk1/2 (p-Erk1/2), anti-Erk1/2, anti-Bcl-2, anti-cleaved Caspase-3, and anti-glyceraldehyde 3-phosphate dehydrogenase, followed by incubation with horseradish peroxidase–conjugated secondary antibody (Supplemental Table S1, http://links.lww.com/SHK/B646). The blots were stained using an ECL Plus kit (Beyotime, China) and visualized using the ECL Imaging System (Tanon, Shanghai, China). Finally, the blots were normalized to anti-glyceraldehyde 3-phosphate dehydrogenase and quantitatively analyzed using the Image J Software (NIH, Bethesda, MD).

### Cytokine analysis

The level of IL-4 in the plasma was measured using a rat IL-4 enzyme-linked immunosorbent assay kit (Bioswamp, Wuhan, China) according to the manufacturer’s protocols. The results are expressed as picograms of the measured cytokine per milliliter of plasma.

### Immunohistochemistry

The spleen tissue sections from four rats in each group were incubated with the following antibodies: p-Akt, p-Erk1/2, Bcl-2, cleaved Caspase-3, and β_1_ adrenoreceptors (Supplemental Table S1, http://links.lww.com/SHK/B646), followed by counterstaining with hematoxylin. Five randomly chosen fields of view were quantified for each section at 400× magnification. As previously described (22), the staining intensity was graded semiquantitatively (0, undetectable; 1, weak; 2, moderate; 3, strong).

### Immunofluorescence

The spleen tissue section of a healthy rat was incubated with rabbit anti-CD4 antibody and anti-β_1_ adrenoreceptor antibody, followed by staining with fluorochrome-conjugated secondary antibodies (Supplemental Table S1, http://links.lww.com/SHK/B646). The section was stained with 4′-6-diamidino-2-phenylindole and then observed under a fluorescence microscope (Olympus, Tokyo, Japan).

### Quantitative real-time polymerase chain reaction

Total RNA of splenic CD4^+^ T-lymphocytes from six rats in each group was extracted. According to the manufacturer’s manual, the purified mRNA from each sample was reverse transcribed into complementary deoxyribonucleic acid (cDNA) using PrimeScript RT Master Mix (Vazyme, Nanjing, China)(Supplemental Table S2, http://links.lww.com/SHK/B646). Quantitative real-time polymerase chain reactions (qRT-PCR) was performed using the UltraSYBR Mixture (CWbio, Beijing, China). The relative mRNA expression level of the β_1_-adrenoreceptor in splenic CD4^+^ T-lymphocytes was calculated using the 2^−ΔΔCt^ method.

### Statistical analysis

Data are expressed as median with interquartile range (IQR) in main text and tables and as median with upper edges of error bars representing the 75th percentile in figures. The Mann-Whitney test was performed to evaluate the differences between the sham and CLP groups. The Kruskal-Wallis test was performed between the CLP, CLP + E-5, and CLP + E-18 groups. When the Kruskal-Wallis test was significant at the 5% level, Dunnett multiple post hoc comparisons were performed. The data were plotted using GraphPad Prism 7.0 software (GraphPad Software, San Diego, CA) and analyzed using IBM-SPSS Statistics 23.0 (IBM Corp, NY).

## RESULTS

### Model characterization

Compared with rats in the sham group, CLP induced arterial hypotension (sham 131 mm Hg [123–136 mm Hg]; CLP 77 mm Hg [71–86 mm Hg], *P* = 0.001), tachycardia (sham 335 bpm [319–352 bpm]; CLP 371 bpm [363–380 bpm], *P* = 0.005) and elevated lactatemia (sham 0.9 mmol·l^−1^ [1.0–1.1 mmol·l^−1^]; CLP 2.4 mmol·l^−1^ [2.1–3.2 mmol·l^−1^], *P* = 0.001; Table 1). The CLP induced (1) cardiac muscle fibers destruction, congestion, and inflammatory infiltration (Supplemental Fig. S4A, http://links.lww.com/SHK/B646); (2) apparent inflammatory cells aggregation, intra-alveolar capillary hemorrhages, and thickening of the alveolar walls in lung tissues (Supplemental Fig. S4B, http://links.lww.com/SHK/B646); and (3) depletion of reticuloendothelial cells and lymphocytes in spleen tissues (Supplemental Fig. S4C, http://links.lww.com/SHK/B646). The heart (*P* = 0.002), lung (*P* = 0.002), and spleen (*P* = 0.002) injury scores were increased in the CLP group (Table 1).

**Table 1:** Comparison of hemodynamics and organ injury score 18 hours after operation in different groups

Variables	Sham	IQR	CLP	IQR	CLP + E-5	IQR	CLP + E-18	IQR	*p*-value	
	Median		Median		Median		Median			
Heart rate(min^-1^)	335	319-352	371	363-380	368	356-389	245	236-267	0.005	CLP *vs.* Sham
									1.000	CLP *vs.* CLP + E-5
									0.001	CLP *vs.* CLP + E-18
Mean arterial pressure (mmHg)	131	123-136	77	71-86	101	98-105	92	80-104	0.001	CLP *vs.* Sham
									0.006	CLP *vs.* CLP + E-5
									0.529	CLP *vs.* CLP + E-18
Lactatemia (mmol.L^-1^)	0.9	1.0-1.1	2.4	2.1-3.2	1.4	1.2-1.7	2.4	2.1-2.6	0.001	CLP *vs.* Sham
									0.046	CLP *vs.* CLP + E-5
									0.785	CLP *vs*. CLP + E-18
heart injury score	0.3	0.20-0.55	2.1	2.0-2.2	1.5	1.25-1.75	1.9	1.5-2.0	0.002	CLP *vs.* Sham
									0.024	CLP *vs.* CLP + E-5
									0.516	CLP *vs*. CLP + E-18
lung injury score	0.4	0.40-0.55	2.3	2.2-2.4	1.6	1.45-1.75	1.9	1.8-2.0	0.002	CLP *vs.* Sham
									0.012	CLP *vs.* CLP + E-5
									0.117	CLP *vs.* CLP + E-18
spleen injury score	0.3	0.05-0.40	2	2.0-2.15	1.8	1.5-1.8	1.8	1.65-1.95	0.002	CLP *vs.* Sham
									0.043	CLP *vs.* CLP + E-5
									0.086	CLP *vs.* CLP + E-18

CLP: cecal ligation and puncture; IQR: interquartile range; CLP + E-5: CLP with Esmolol infused at 5 mg.kg^−1^.h^−1^; CLP + E-18: CLP with Esmolol infused at 18 mg.kg^−1^.h^−1^

### Effects of different doses of esmolol on hemodynamics and organ injuries

Compared with rats in the CLP group, esmolol infused at 5 mg·kg^−1^·h^−1^ did not reduce HR (CLP 371 bpm [363–380 bpm]; CLP + E-5 368 bpm [356–389 bpm], *P* = 1.000), but restored MAP (CLP 77 mm Hg [71–86 mm Hg]; CLP + E-5 101 mmHg [98–105 mm Hg], *P* = 0.006) and decreased the circulating level of lactate (CLP 2.4 mmol·l^−1^ [2.1–3.2 mmol·l^−1^]; CLP + E-5 1.4 mmol·l^−1^ [1.2–1.7 mmol·l^−1^], *P* = 0.046; Table 1). Compared with rats in the CLP group, esmolol infused at 18 mg·kg^−1^·h^−1^ reduced HR (CLP 371 bpm [363–380 bpm]; CLP + E-18 245 bpm [236–267 bpm], *P* = 0.001), but did not worsen MAP (CLP 77 mm Hg [71–86 mm Hg]; CLP + E-18 92 mm Hg [80–104 mm Hg], *P* = 0.529), and had no effects on circulating level of lactate (CLP 2.4 mmol·l^−1^ [2.1–3.2 mmol·l^−1^]; CLP + E-18 2.4 mmol·l^−1^ [2.1–2.6 mmol·l^−1^], *P* = 0.785; Table 1). Infusion of esmolol at 5 mg·kg^−1^·h^−1^ improved CLP induced (1) cardiac muscle fibers destruction, congestion, and inflammatory infiltration (Supplemental Fig. S4A, http://links.lww.com/SHK/B646); (2) inflammatory cells aggregation, intra-alveolar capillary hemorrhages, and thickening of the alveolar walls in lung tissues (Supplemental Fig. S4B, http://links.lww.com/SHK/B646); and (3) depletion of reticuloendothelial cells and lymphocytes in spleen tissues (supplemental Fig. S4C, http://links.lww.com/SHK/B646). Compared with the CLP group, the heart (*P* = 0.024), lung (*P* = 0.012), and spleen (*P* = 0.043) injury scores were reduced in the CLP + E-5 group (Table 1). Infusion of esmolol at 18 mg·kg^−1^·h^−1^ did not significantly improve CLP-induced heart, lung, and spleen injuries (heart: *P* = 0.516; lung: *P* = 0.117; spleen: *P* = 0.086; Table 1).

### Eighteen-hour mortality

Eighteen hours after CLP, the mortality rate was 0 of 8, 4 of 12, 1 of 9, and 2 of 10, respectively, in sham, CLP, CLP + E-5, and CLP + E-18 group (Supplemental Table S3, http://links.lww.com/SHK/B646).

### Effects of different doses of esmolol on PBMCs apoptosis

**Fig. 2:**
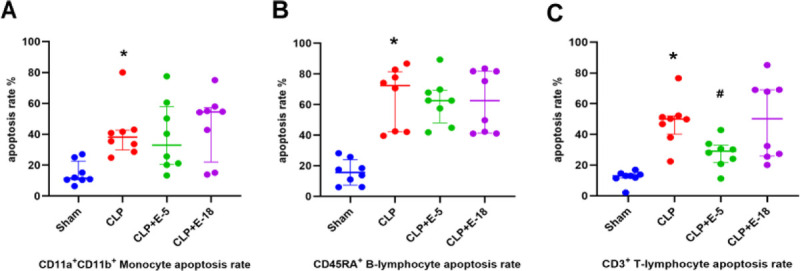
**Effects of different doses of esmolol on apoptosis of PBMCs by flow cytometry.** Apoptosis rates of circulating monocytes (A), B-lymphocytes (B), and T-lymphocytes (C) in different groups were shown in the histogram. Data are expressed as median ± interquartile range, n = 8. The upper edges of error bars represent the 75th percentile in each group. *P < 0.05: CLP group vs. sham group; #*P* < 0.05: CLP + E-5 vs. CLP group. CLP, cecal ligation and puncture; CLP + E-5, CLP with esmolol infused at 5 mg·kg^−1^·h^−1^; CLP + E-18, CLP with esmolol infused at 18 mg·kg^−1^·h^−1^; PBMCs, peripheral blood mononuclear cell.

Compared with the sham group, CLP increased the apoptosis of monocytes (*P* = 0.001), B-lymphocytes (*P* = 0.001), and T-lymphocytes (*P* = 0.001; Fig. 2). Infusion of esmolol at both 5 and 18 mg·kg^−1^·h^−1^ did not reduce CLP-induced apoptosis of monocytes (CLP; vs. CLP + E-5, *P* = 0.993; vs. CLP + E-18, *P* = 0.934; Fig. 2A) and B-lymphocytes (CLP; vs. CLP + E-5, *P* = 0.989; vs. CLP + E-18, *P* = 0.992; Fig. 2B). Infusion of esmolol at 5 mg·kg^−1^·h^−1^ significantly reduced apoptosis of T-lymphocytes (*P* = 0.019; Fig. 2C). However, infusion of esmolol at 18 mg·kg^−1^·h^−1^ did not significantly reduced apoptosis of T-lymphocytes (*P* = 0.999; Fig. 2C).

### Effects of different doses of esmolol on T_H_0 differentiation

**Fig. 3:**
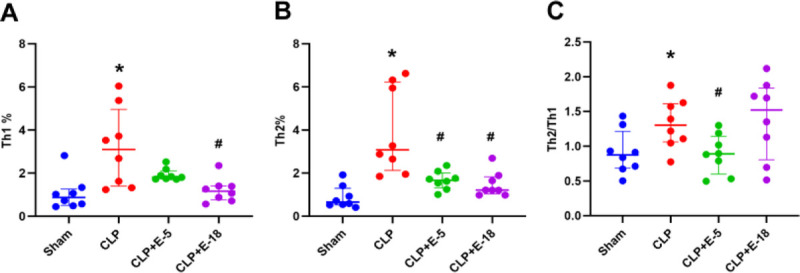
**Effects of different doses of esmolol on T-lymphocyte subsets by flow cytometry.** The percentages of T_H_1 (A) and T_H_2 (B) in T helper cells and the ratio of T_H_2/T_H_1 (C) were shown in the histogram. Data are expressed as median ± interquartile range, n = 8. The upper edges of error bars represent the 75th percentile in each group. * *P* < 0.05: CLP group vs. sham group; # *P* < 0.05: CLP + E-5, CLP + E-18 vs. CLP group. CLP, cecal ligation and puncture; CLP + E-5, CLP with esmolol infused at 5 mg·kg^−1^·h^−1^; CLP + E-18, CLP with esmolol infused at 18 mg·kg^−1^·h^−1^.

The CLP induced an increase in both T_H_1(*P* = 0.005) and T_H_2 (*P* = 0.001) levels compared with the sham group (Fig. 3A and B). Infusion of esmolol at both 5 and 18 mg·kg^−1^·h^−1^ decreased CLP-induced increase of T_H_2 (CLP; vs. CLP + E-5, *P* = 0.042; vs. CLP + E-18, *P* = 0.029), but only at 18 mg·kg^−1^·h^−1^ attenuated CLP-induced increase of T_H_1 (CLP; vs. CLP + E-5, *P* = 0.232; vs. CLP + E-18, *P* = 0.049). Thus, infusion of esmolol at 5 mg·kg^−1^·h^−1^ rather than 18 mg·kg^−1^·h^−1^ lowered the ratio of T_H_2 to T_H_1 (CLP; vs. CLP + E-5, *P* = 0.049; vs. CLP + E-18, *P* = 0.992; Fig. 3C).

### Effects of different doses of esmolol on apoptosis-associated signaling proteins

**Fig. 4:**
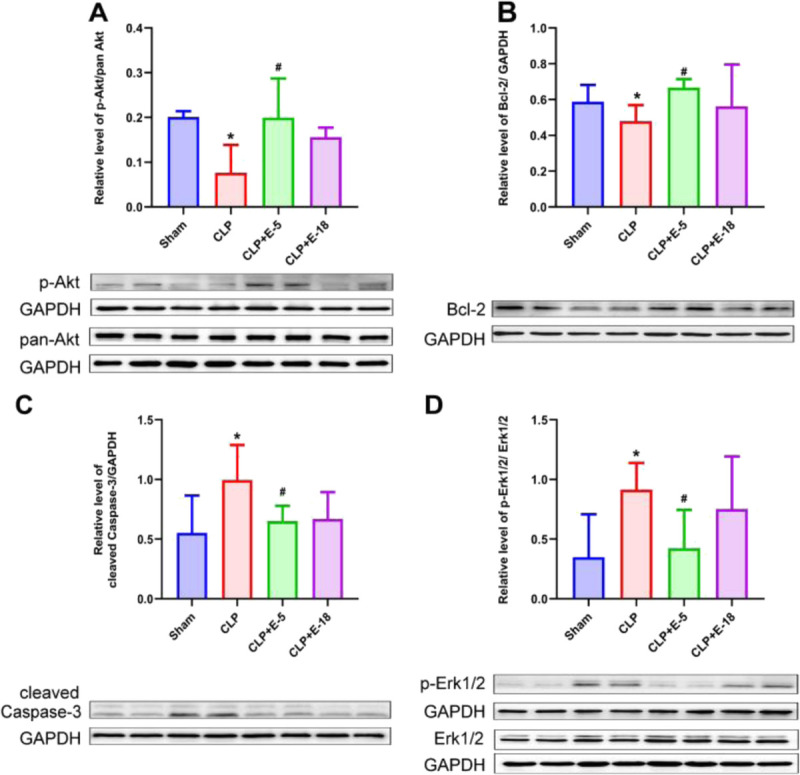
**Effects of different doses of** **esmolol** **on apoptosis-associated signaling proteins and Th0 differentiation-associated signaling protein by western blots.** Western blots revealed p-Akt (A), Bcl-2 (B), cleaved Caspase-3 (C), and p-Erk1/2 (D). Proteins were obtained from splenic CD4^+^ T-lymphocytes lysates (n = 8) prepared from all experimental rat groups. Two typical western blots are shown below each histogram. Densitometric analysis was used to calculate the normalized protein ratio. Data are expressed as median ± interquartile range. The upper edges of error bars represent the 75th percentile in each group. * *P* < 0.05: CLP group vs. sham group; # *P* < 0.05: CLP + E-5 vs. CLP group. CLP, cecal ligation and puncture; CLP + E-5, CLP with esmolol infused at 5 mg·kg^−1^·h^−1^; CLP + E-18, CLP with esmolol infused at 18 mg·kg^−1^·h^−1^. p-Akt phosphorylated Akt; p-Erk1/2, phosphorylated Erk1/2.

Immunofluorescence analysis confirmed that β_1_-adrenoreceptors were expressed on the surface of splenic CD4^+^ T-lymphocytes (Supplemental Fig. S5, http://links.lww.com/SHK/B646). Tested by western blot, CLP decreased the expression of p-Akt (*P* = 0.010) and Bcl-2 (*P* = 0.049) and increased cleaved Caspase-3 (*P* = 0.001) compared with the sham group (Fig. 4A–C). Infusion of esmolol at 5 mg·kg^−1^·h^−1^ increased the expression of p-Akt (*P* = 0.048) and Bcl-2 (*P* = 0.032) and decreased cleaved Caspase-3 (*P* = 0.048) compared with the CLP group (Fig. 4A–C). However, infusion of esmolol at 18 mg·kg^−1^·h^−1^ did not increase expression of p-Akt (*P* = 0.250) and Bcl-2 (*P* = 0.256), which had a tendency to decrease cleaved Caspase-3 (*P* = 0.054) compared with CLP group (Fig. 4A–C). Then, immunohistochemistry confirmed the results of western blot (sham vs. CLP: p-Akt, *P* = 0.029; Bcl-2, *P* = 0.029; cleaved Caspase-3, *P* = 0.029; CLP vs. CLP + E-5: p-Akt, *P* = 0.009; Bcl-2, *P* = 0.033; cleaved Caspase-3, *P* = 0.027; CLP vs.CLP + E-18: p-Akt, *P* = 0.698; Bcl-2, *P* = 0.628; cleaved Caspase-3, *P* = 0.996) (Supplemental Fig. S6A, B and C, http://links.lww.com/SHK/B646).

### Effects of different doses of esmolol on T_H_0 differentiation-associated signaling proteins

**Fig. 5:**
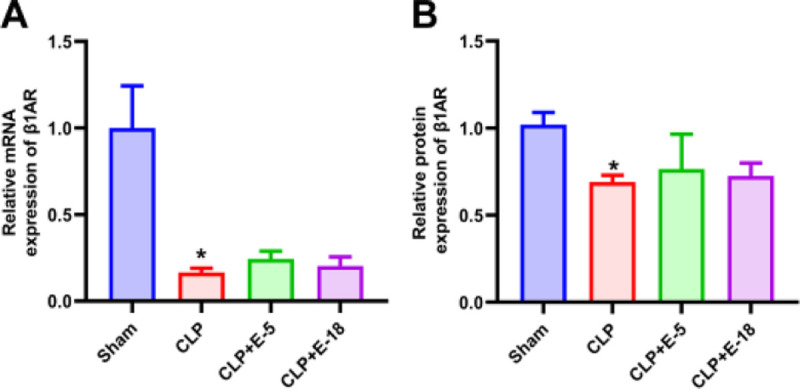
**Effects of esmolol on mRNA and protein expression of β**_1_**-adrenoceptors on splenic CD4**^+^ **T-lymphocytes**. mRNA expression levels of β_1_-adrenoceptor (A) were evaluated by quantitative real-time polymerase chain reaction (n = 6). The results were first normalized to one housekeeping gene and thereafter to sham expression, which was set at 1. Immunohistochemistry revealed β_1_-adrenoceptor in the splenic tissues. The staining intensity was used to evaluate β_1_-adrenoceptor (B) expression level (n = 4). Data are expressed as median ± interquartile range. The upper edges of error bars represent the 75th percentile in each group. *P < 0.05, CLP group vs. sham group. CLP, cecal ligation and puncture; CLP + E-5, CLP with esmolol infused at 5 mg·kg^−1^·h^−1^; CLP + E-18, CLP with esmolol infused at 18 mg·kg^−1^·h^−1^.

Compared with the sham group, CLP increased Erk1/2 phosphorylation analyzed by western blot (*P* = 0.015; Fig. 4D). Infusion of esmolol at 5 mg·kg^−1^·h^−1^ reduced CLP-induced increased in Erk1/2 phosphorylation (*P* = 0.009; Fig. 4D). However, infusion of esmolol at 18 mg·kg^−1^·h^−1^ did not significantly decrease the CLP-induced increased in Erk1/2 phosphorylation (*P* = 0.992; Fig. 5A). The results were confirmed by immunohistochemistry (CLP vs. sham, *P* = 0.029; vs. CLP + E-5, *P* = 0.041; vs. CLP + E-18, *P* = 0.223; Supplemental Fig. S6D, http://links.lww.com/SHK/B646).

### Effect of different doses of esmolol on circulatory IL-4 level

Compared with the sham group, CLP was associated with increased plasma levels of IL-4 (*P*<0.001)(Supplemental Fig. S7, http://links.lww.com/SHK/B646). Addition of esmolol at 5 mg·kg^−1^·h^−1^ in CLP rats resulted in a decrease in plasma IL-4 level (*P* = 0.031). However, there were no significant difference in plasma IL-4 level between the CLP and CLP + E-18 groups (*P* = 0.837).

### Effects of different doses of esmolol on the β_1_-adrenoreceptor expression on T-lymphocytes

Compared with the sham group, CLP decreased mRNA (*P* = 0.002) and protein (*P* = 0.029) expression of β_1_-adrenoreceptor in splenic CD4^+^ T-lymphocytes tested by qRT-PCR and immunohistochemistry (Fig. 5 and Supplemental Figure S8, http://links.lww.com/SHK/B646). Infusion of esmolol at both 5 and 18 mg·kg^−1^·h^−1^ did not modulate the mRNA (CLP vs. CLP + E-5, *P* = 0.669; vs. CLP + E-18, *P* = 0.869; Fig. 5A) and protein (CLP vs. CLP + E-5, *P* = 0.352; vs. CLP + E-18, *P* = 0.824; Fig. 5B) expression of β_1_-adrenoreceptor in splenic CD4^+^ T-lymphocytes.

## DISCUSSION

The main result of the study is that blocking β_1_-adrenoreceptors by esmolol decreased circulating T-lymphocyte apoptosis and restored peripheral blood T_H_2/T_H_1 ratio in septic shock model. Akt/Bcl-2/Caspase-3 pathway, which was associated with T-lymphocyte apoptosis, was found to be modulated by esmolol. Erk1/2 activity, which promoted T_H_0 differentiation to T_H_2, was revealed to be inhibited by esmolol. Esmolol at low dose without heart rate reduction showed better immunomodulatory effects than at high dose with heart rate reduction.

### Model characteristics

In the study, the CLP model was used to establish the septic shock model. All rats were resuscitated with adapted fluids. Antibiotics was infused 4 hours after CLP to mimic clinical settings. As in previous studies (9,10,20,23), all rats that underwent CLP showed the typical characteristics of septic shock, including hypotension, hyperlactatemia, and multiple organ injuries, including heart, lung, and spleen (Table 1 and Supplemental Fig. S4, http://links.lww.com/SHK/B646).

### Effects of esmolol on survival

Ackland et al. (24) pretreated sepsis rats with β_1_-adrenoreceptor blockers (metoprolol and atenolol) 2 days before injection of LPS improved survival. Metoprolol increased median time to death in sepsis rats when pretreated 2 days before CLP. However, both metoprolol and atenolol failed to improve survival when treatment commenced 6 hours after induction of sepsis in their study. The doses used of metoprolol and atenolol resulted in a 20% reduction in the heart rate from baseline in the study. Medical treatment is usually after sepsis insult in clinical settings. To reproduce clinical setting of septic patients, the following studies commenced treatment after sepsis insult. Mori et al. (25) administrated septic rats with esmolol infusion 1 hour after CLP. The dose of esmolol also reduced heart rate by approximately 20% as compared with baseline. They found the survival time was significantly improved in esmolol group. Kimmoun et al. (9) infused septic rats with esmolol 4 hours after CLP with a dose reducing heart rate as compared with CLP group. Median time to death was also increased in esmolol-treated septic rats. In contrast to previous studies, Ibrahim-zada et al. (26) infused sepsis rats with esmolol 4 hours after injection of LPS with a very low dose without any effect on myocardial function and also showed survival improvement in esmolol group. Previous results showed that both high and low doses of β_1_-adrenoreceptor blockers improved survival in experimental sepsis. In our study, the 18-hour mortality was 33.3%, 11.1%, and 20%, respectively, in CLP, CLP + E-5, and CLP + E-18 group, which was consistent with previous studies (9,24–26).

### Effects of esmolol on T-lymphocyte apoptosis

Previous studies have reported that immunosuppression predominately results from apoptosis of monocytes, B-lymphocytes, and T-lymphocytes in septic shock patients (3,27). In our study, CLP induced apoptosis of circulating monocytes, B-lymphocytes, and T-lymphocytes, which was similar to the clinical settings. Overstimulation of immune cells *via* adrenergic receptors by catecholamines, which are secreted by the sympathetic nervous system overactivation, contributes to their apoptosis in septic shock (28,29). Our results showed that blocking β_1_-adrenergic receptors by esmolol at low dose significantly reduced T-lymphocyte apoptosis in rats with septic shock.

### Mechanisms of esmolol on T-lymphocyte apoptosis

β_1_-Adrenoreceptor could modulate Akt *via* Gβγ/PI3K (13). Our previous study showed that blocking β_1_-adrenoreceptors by esmolol could increase Akt phosphorylation (10). Akt activation leads to Bad phosphorylation resulting in Bcl-2 release, ultimately promoting cell survival (14). Our results revealed that low dose of esmolol increased Akt phosphorylation and Bcl-2 expression and reduced cleaved Caspase-3 in splenic CD4^+^ T-lymphocytes in septic shock models. The results were consistent with the report by Qi et al. (15) in cardiomyocytes that phosphorylation of Akt induced upregulation of Bcl-2 and downregulation of cleaved Caspase-3. Therefore, esmolol might reduce T-lymphocytes apoptosis in septic shock by modulating the Akt/Bcl-2/Caspase-3 pathway (Fig. 1).

### Effects of esmolol on T_H_2/T_H_1 ratio

Previous studies have shown an imbalance of T-lymphocyte subpopulations in septic patients, such as an augmented T_H_2/T_H_1 ratio (5,30,31). Our results showed an increased peripheral blood T_H_2/T_H_1 ratio in septic shock models as clinical settings. Infusion of esmolol at low dose restored the ratio of T_H_2 and T_H_1 in septic shock.

### Mechanisms of esmolol on T_H_2 differentiation

Induction of T_H_0 into the T_H_2 differentiation pathway depends to a significant extent on IL-4 produced upon TCR cross-linking (32). Interleukin 4 establishes a positive feedback loop through IL-4R, which further reinforces IL-4 expression while silencing the IFN-γ locus at the same time in T_H_0 (18,19). Erk influences TCR-dependent activation of IL-4 gene transcription through association to the proximal promoter. Thus, Erk modulates T_H_0 differentiation *via* TCR-dependent IL-4 production. Activation of the β_1_-adrenoreceptor could induce Erk phosphorylation through the adenylyl cyclase–cyclic adenosine monophosphate–protein kinase A pathway (16). Our results showed that blocking β_1_-adrenoreceptor by esmolol decreased CLP-induced Erk1/2 phosphorylation. Circulating IL-4 level was also decreased in esmolol-treated group, which was consistent with the study by Manon et al. (11). In their study, blocking β_1_-adrenoreceptor by esmolol decreased circulating IL-4 level in septic shock mice. These findings supported that esmolol might reduce T_H_0 differentiation to T_H_2 in septic shock by decreasing IL-4 production *via* inhibiting of Erk1/2 activation (Fig. 1).

### Different doses of esmolol on immunomodulation

The optimal dose of esmolol for immunoregulation of septic shock remains unclear. Our results showed that esmolol at low dose without heart rate reduction rather than at high dose with heart rate reduction significantly decreased T-lymphocytes apoptosis and restored T_H_2/T_H_1 ratio in septic shock models. Thus, low dose of esmolol might be more promising for modulating the immune response in septic shock. More studies are needed to confirm our findings.

### Effects of esmolol on β_1_-adrenoreceptor on T-lymphocytes

In septic shock, excessive stimulation by catecholamine resulted in the reduction of β_1_-adrenoreceptor density in cardiomyocytes, which was restored by esmolol treatment (6). Our results demonstrated that the mRNA and protein expression of β_1_-adrenoreceptor also decreased in T-lymphocytes in septic shock rats. However, esmolol infusion at different doses did not restore the mRNA or protein expression of β_1_-adrenoreceptor on T-lymphocytes in septic shock rats. The effects of esmolol on its receptor expression may vary in different cells. Further study is needed to elucidate the mechanisms.

### Study limitation

In fact, esmolol also decreased peripheral blood T_H_0 differentiation to T_H_1 in septic shock in the study. However, the mechanisms were not explored in the study. Our work is just a starting point for investigating the effects and mechanisms of esmolol on T_H_0 differentiation. More follow-up work is needed. Besides, the immune status varies during the course of septic shock. The optimal time to the initiation of esmolol treatment requires future investigation. Lastly, only male rats were used in this study. Female rats should be used in the following researches to complete date for the entire population.

## CONCLUSIONS

Esmolol reduced circulating T-lymphocyte apoptosis and restored the peripheral blood T_H_2/T_H_1 ratio. Esmolol might modulate the Akt/Bcl-2/Caspase-3 pathway to relieve T-lymphocyte apoptosis and inhibit Erk1/2 activity to decrease peripheral blood T_H_0 differentiation to T_H_2. Esmolol at low dose without heart rate reduction showed better immunomodulatory effects than at high dose. Esmolol at low dose may be a potential immunoregulator of septic shock.

## Supplementary Material

SUPPLEMENTARY MATERIAL
