# Supplementary material for: LOW DOSE OF ESMOLOL ATTENUATES SEPSIS-INDUCED IMMUNOSUPPRESSION VIA MODULATING T-LYMPHOCYTE APOPTOSIS AND DIFFERENTIATION
Source: Shock. 2023 Feb 28;59(5):771–8. doi: 10.1097/SHK.0000000000002104 (PMC10125111; doi:10.1097/SHK.0000000000002104)
Supplement: SUPPLEMENTARY MATERIAL [file shock-59-771-s001.docx]

**Low dose of Esmolol attenuates sepsis-induced immunosuppression via modulating T-lymphocyte apoptosis and differentiation**

Ying Ma ^1,2^, Zhenshun Cheng ^1,2,4^, Yong Zheng ^3^, Wei Wang ^1^, Shaojun He ^1^, Xiaolian Zhou ^1^, Jiong Yang ^1^, Chaojie Wei ^1,2^

1. Department of Pulmonary and Critical Care Medicine, Zhongnan Hospital of Wuhan University, Wuhan, 430071, Hubei, China
2. Wuhan Research Center for Infectious Diseases and Cancer, Chinese Academy of Medical Sciences, Wuhan, 430071, Hubei, China
3. Department of Anatomy and Embryology, Wuhan University Taikang Medical School (School of Basic Medical Sciences), Wuhan, 430071, Hubei, China
4. Hubei Engineering Center for Infectious Disease Prevention, Control and Treatment, Wuhan, 430071, Hubei, China

**Corresponding author information:**

Corresponding author 1: Chaojie Wei, Department of Pulmonary and Critical Care Medicine, Zhongnan Hospital of Wuhan University, Wuhan, 430071, China. E-mail: chaojie_wei@163.com

Corresponding author 2: Jiong Yang, Department of Pulmonary and Critical Care Medicine, Zhongnan Hospital of Wuhan University, Wuhan, 430071, China. E-mail: yangjiongwh@126.com

**Funding**

This work was kindly supported by the National Natural Science Foundation of China (81801961), the Non-profit central research institute fund of the Chinese Academy of medical sciences (2020-PT320-004), and the climbing project for the medical talent of Zhongnan Hospital, Wuhan University (PDJH202205).

**Abstract**

**Background:** Immunosuppression caused by immune cell apoptosis and an imbalance of T help 2 cells (Th2) and T help 1 cells (Th1), is associated with poor outcomes in septic patients. Esmolol was reported to improve survival by modulating immune responses in septic shock. Whether Esmolol could alleviate sepsis-induced immunosuppression and the optimal dose are unclear.

**Methods:** Four hours after cecal ligation and puncture (CLP), Wistar rats were randomized into CLP, CLP+E-5 (Esmolol:5mg.kg^-1^.h^-1^) and CLP+E-18 (Esmolol:18mg.kg^-1^.h^-1^) groups. Eight rats were underwent sham operation. Eighteen hours after CLP, hemodynamics and organ histological injuries were evaluated, peripheral blood mononuclear cells apoptosis and T-lymphocyte subsets counts were determined by flow cytometry, and the expression of p-Akt, Bcl-2, cleaved Caspase-3 and p-Erk1/2 in splenic CD4^+^ T-lymphocytes was determined by western blot and immunohistochemistry. β1-adrenoreceptor expressions were evaluated using real-time PCR and immunohistochemistry.

**Results:** CLP induced tachycardia, hypotension, hyperlactatemia and multiple organ injury. Heart rate was unchanged in the CLP+E-5 group but decreased in the CLP+E-18 group. Hypotension, lactatemia and multiple organ injuries were improved only in the CLP+E-5 group. T-lymphocyte apoptosis and Th2/Th1 ratio was decreased in CLP+E-5 but not in CLP+E-18. p-Akt and Bcl-2 expressions were increased, while cleaved Caspase-3 and p-Erk1/2 expressions were decreased in CLP+E-5. β1-adrenoreceptor expressions were unchanged in both CLP+E-5 and CLP+E-18 groups.

**Conclusions:** Low dose of Esmolol reduced T-lymphocyte apoptosis and restored Th2/Th1 ratio in septic shock. Esmolol might modulate Akt/Bcl-2/Caspase-3 pathway to relieve T-lymphocyte apoptosis and inhibit Erk1/2 activity to decrease Th0 differentiation to Th2. Esmolol may be a potential immunoregulator of septic shock.

**Keywords:** septic shock, Esmolol, T-lymphocyte, apoptosis, differentiation

**Introduction**

Septic shock develops as a dysregulated host inflammatory response to infection, resulting in multiple organ dysfunction that is associated with high mortality worldwide(1). Both pro-and anti-inflammatory immune responses occur after the onset of sepsis. If sepsis persists, patients will enter a markedly immunosuppressive state(2). Immunosuppressed septic patients are at a high risk of secondary nosocomial infection resulting in an increasing 13% mortality of septic patients(3). Immune cell apoptosis, such as circulating monocytes, B-lymphocytes and T-lymphocytes, and up-regulated T helper 2 cells (Th2)/T helper 1 cells (Th1) ratio are the most reported characteristics contributing to immunosuppression during septic shock(4, 5).

In experimental and clinical studies, Esmolol, a highly selective ultra-short-acting β1-adrenoreceptor blocker, was recently reported to improve cardiovascular function and survival in septic shock(6-8). The beneficial effects of Esmolol in septic shock have been previously considered because of its hemodynamic effects(9). However, recent evidence has shown that the beneficial effects of Esmolol in septic shock are associated with immunomodulation(10, 11). However, how Esmolol influences the immune response in septic shock and the optimal dose are unclear.

Lymphocytes and monocytes expressed β1-adrenoreceptors(12), which belong to the G-protein-coupled receptor(GPCR) super-family. GPCRs couple to a G protein heterotrimer including the α, β and γ subunits in the intracellular region (Figure 5). G protein α (Gα) binds to the G protein βγ dimer (Gβγ) in the inactive state. GPCR activation leads to the dissociation of Gα and Gβγ. Gβγ modulates protein kinase B (Akt) via phosphatidylinositide 3-kinases (PI3K)(13). Akt activation leads to B-cell leukemia/lymphoma 2 asociated death promoter (Bad) phosphorylation, resulting in B-cell leukemia/lymphoma 2 (Bcl-2) release, ultimately promoting cell survival(14)*.* Our previous study showed that blocking the β1-adrenoreceptor by Esmolol could increase Akt phosphorylation in cardiovascular tissue(10). Qi *et al.* reported that Akt phosphorylation decreased sepsis-induced cardiomyocyte apoptosis *via* up-regulation of Bcl-2 and down-regulation of cleaved Caspase-3 both in vitro and in vivo(15). Thus, we aimed to determine whether Esmolol could reduce sepsis-induced peripheral blood mononuclear cell (PBMC) apoptosis by modulating Akt/Bcl-2/Caspase-3 pathway. Activated Gα induces extracellular regulated protein kinases (Erk) phosphorylation through the adenylyl cyclase (AC)-cyclic adenosine monophosphate (cAMP)-protein kinase A (PKA) pathway(16). Furthermore, Erk is an obligatory mediator of the Th2 differentiation pathway(17). Interleukin (IL)-4, endogenously produced upon TCR cross-linking, establishes a positive feedback loop through IL-4R that further reinforces IL4 expression in Th0(18, 19), inducing Th0 differentiation to Th2. Erk promotes Th2 differentiation by activating the early phase of TCR-dependent IL-4 production(17)*.* Hence, we also aimed to determine whether Esmolol could reduce Th0 differentiation to Th2 by inhibiting Erk1/2 activation.

**Methods**

**Animals**

Adult male Wistar rats weighing 300–400g were obtained from the Center for Animal Experiments of Wuhan University. All animal experiments were approved by the Institutional Animal Care and Use Committee of the Animal Experiment Center of Wuhan University (E2020072901) and followed the institutional and national guidelines.

**Study design**

The cecal ligation and puncture (CLP) model was used to develop a septic shock model as previously described (20). Four hours after CLP, all rats were randomized into three groups (CLP (n=8), CLP+E-5 (CLP with Esmolol infused at 5mg.kg^−1^.h^−1^ (n = 8)), CLP+E-18 (CLP with Esmolol infused at 18mg.kg^−1^.h^−1^ (n=8)). Eight rats were under sham operation. Four hours after surgery, all rats received fluid resuscitation (Saline 10ml.kg^-1^.h^-1^), antibiotic (Meropenem 10mg.kg^-1^) and analgesic (Nalbuphine 0.2mg.kg^-1^.h^-1^) for 14 hours. The rats in the CLP+E-5 and CLP+E-18 groups received the infusion of Esmolol initiated 4 hours after surgery for a period of 14 hours. Assessments were performed 18 hours after the CLP or sham surgery. 18 hours-mortality was collected in each group.

**Dose selection of Esmolol**

A high dose with heart rate reduction and a low dose without heart rate reduction were chosen in the study to determine the optimal dose. Infusing septic shock rats with Esmolol at 18 kg^-1^.h^-1^ induced heart rate reduction compared to CLP group in the previous study(10). To compare with the previous study, 18mg.kg^-1^.h^-1^ was chosen as the high dose with heart rate reduction in the study. The low dose was chosen according to our previous study(10). In this study, we did a dose gradient analysis (Esmolol infused at 1mg.kg^-1^.h^-1^, 5mg.kg^-1^.h^-1^ and 18mg.kg^-1^.h^-1^) and found that Esmolol infused at both 5 and 1mg.kg^-1^.h^-1^ did not reduce heart rate but 5mg.kg^-1^.h^-1^ presented better immunomodulatory effects compared to 1mg.kg^-1^.h^-1^. Thus, Esmolol infused at 5mg.kg^-1^.h^-1^ was chosen as the low dose without heart rate reduction.

**Hemodynamics and organ injuries measurement**

The pressure transducer catheter was inserted into the right carotid artery of anesthetized rats and connected to a BL-420N biological signal recorder (Taimeng, Chengdu, China) to measure the HR and MAP. Arterial blood was collected for lactate detection using an ABL800 FLEX blood-gas Analyser™ (Radiometer, Denmark). Heart, lung and spleen tissue sections were stained with hematoxylin and eosin (H&E) and morphological changes were observed under the optical microscope (Olympus, Tokyo, Japan) at 200×magnification. Histopathological lesions were quantified using five randomly selected fields per slide(21).

**Flow cytometry**

PBMCs were isolated from all rats using Ficoll-Paque (Cytiva, Sweden). Cell surface markers were used to identify circulating monocytes (CD11a^+^CD11b^+^), B-lymphocytes (CD45RA^+^), T-lymphocytes (CD3^+^) and CD4^+^ T-lymphocytes (CD3^+^CD4^+^) in the PBMCs**.** According to the manufacturer's protocol, apoptosis detection was performed using a FITC Annexin V Apoptosis Detection Kit with 7-amino-actinomycin D (7-AAD)**.** After stimulation with the leukocyte activation cocktail, CD4^+^ T-lymphocytes were stained with interferon (INF)-γ and IL-4 to identify Th1 and Th2 counts respectively**.** Antibody and gating information were shown in Supplemental Table S1 and Supplemental Figure S1 and S2, http://links.lww.com/SHK/B646.

**Western blot**

Due to the limited numbers of circulating T-lymphocytes, splenic CD4^+^ T-lymphocytes were used. Splenic CD4^+^ T-lymphocytes were purified using Rat CD4 microbeads (Miltenyi Biotec, Bergisch Gladbach, Germany) following the manufacturer's instructions. The purity of splenic CD4^+^ T-lymphocytes was determined by flow cytometry (Beckman Coulter, Indianapolis IN, USA) using PE/CY7-labeled anti-CD4 antibody (Supplemental Table S1 and Supplemental Figure S3, http://links.lww.com/SHK/B646).

Protein was extracted from isolated splenic CD4^+^ T-lymphocytes from all rats. After separation on an SDS-PAGE gel, the protein samples were transferred to a PVDF membrane (Millipore, USA) and incubated with the following antibodies: anti-phosphorylated-Akt (p-Akt), anti-Akt, anti-phosphorylated-Erk1/2 (p-Erk1/2), anti-Erk1/2, anti-Bcl-2, anti-cleaved Caspase-3, and anti-Glyceraldehyde 3-phosphate dehydrogenase (GAPDH), followed by incubation with horseradish peroxidase (HRP)-conjugated secondary antibody (Supplemental Table S1, http://links.lww.com/SHK/B646). The blots were stained using an ECL Plus kit (Beyotime, China) and visualized using the ECL Imaging System (Tanon, Shanghai, China). Finally, the blots were normalized to GAPDH and quantitatively analyzed using the Image J Software (NIH, Bethesda, MD, USA).

**Cytokine Analysis**

The level of IL-4 in the plasma was measured using a rat IL-4 enzyme-linked immunosorbent assay (ELISA) kit (Bioswamp, Wuhan, China) according to the manufacturer's protocols. The results are expressed as picograms of the measured cytokine per milliliter of plasma.

**Immunohistochemistry**

The spleen tissue sections from four rats in each group were incubated with the following antibodies: p-Akt, p-Erk1/2, Bcl-2, cleaved Caspase-3 and β1 adrenoreceptors (Supplemental Table S1, http://links.lww.com/SHK/B646), followed by counterstaining with hematoxylin. Five randomly chosen fields of view were quantified for each section at 400×magnification. As previously described(22), the staining intensity was graded semi-quantitatively (0, undetectable; 1, weak; 2, moderate; 3, strong).

**Immunofluorescence**

The spleen tissue section of a healthy rat was incubated with rabbit anti-CD4 antibody and anti-β1 adrenoreceptor antibody, followed by staining with fluorochrome-conjugated secondary antibodies (Supplemental Table S1, http://links.lww.com/SHK/B646). The section was stained with 4’-6-diamidino-2-phenylindole (DAPI) and then observed under a fluorescence microscope (Olympus, Tokyo, Japan).

**Quantitative real-time PCR (qRT-PCR)**

Total RNA of splenic CD4^+^ T-lymphocytes from six rats in each group was extracted. According to the manufacturer's manual, the purified mRNA from each sample was reverse transcribed into complementary deoxyribonucleic acid (cDNA) using PrimeScript RT Master Mix (Vazyme, Nanjing, China)(Supplemental Table S2, http://links.lww.com/SHK/B646). qRT-PCRs was performed using the UltraSYBR Mixture (CWbio, Beijing, China). The relative mRNA expression level of the β1-adrenoreceptor in splenic CD4^+^ T-lymphocytes was calculated using the 2-^ΔΔCt^ method.

**Statistical analysis**

Data are expressed as median with interquartile range (IQR) in main text and tables and as median with upper edges of error bars representing the 75th percentile in figures. The Mann-Whitney test was performed to evaluate the differences between the sham and CLP groups. The Kruskal-Wallis test was performed between the CLP, CLP+E-5 and CLP+E-18 groups. When the Kruskal-Wallis test was significant at the 5% level, Dunnett's multiple post-hoc comparisons were performed. The data were plotted using GraphPad Prism 7.0 software (GraphPad Software, San Diego, CA) and analyzed using IBM-SPSS Statistics 23.0 (IBM Corp., NY, USA).

**Results**

**Model characterization**

Compared to rats in the sham group, CLP induced arterial hypotension (sham 131mmHg(123-136mmHg); CLP 77mmHg(71-86mmHg), *p*=0.001), tachycardia (sham 335bpm(319-352bpm); CLP 371bpm(363-380bpm), *p*=0.005) and elevated lactatemia (sham 0.9mmol.l^-1^(1.0-1.1mmol.l^−1^); CLP 2.4mmol.l^−1^(2.1-3.2mmol.l^−1^), *p*=0.001)(Table 1). CLP induced 1) cardiac muscle fibers destruction, congestion and inflammatory infiltration (Supplemental Figure S4A, http://links.lww.com/SHK/B646); 2) apparent inflammatory cells aggregation, intra-alveolar capillary hemorrhages and thickening of the alveolar walls in lung tissues (Supplemental Figure S4B, http://links.lww.com/SHK/B646); and 3) depletion of reticuloendothelial cells and lymphocytes in spleen tissues (Supplemental Figure S4C, http://links.lww.com/SHK/B646). The heart (*p*=0.002), lung (*p*=0.002) and spleen (*p*=0.002) injury scores were increased in the CLP group (Table 1).

**Effects of different doses of Esmolol on hemodynamics and organ injuries**

Compared to rats in the CLP group, Esmolol infused at 5mg.kg^-1^.h^-1^ didn't reduce HR (CLP 371bpm(363-380bpm); CLP+E-5 368bpm(356-389bpm), *p*=1.000), but restored MAP (CLP 77mmHg(71-86mmHg); CLP+E-5 101mmHg(98-105mmHg), *p*=0.006) and decreased the circulating level of lactate (CLP 2.4mmol.l^−1^(2.1-3.2mmol.l^−1^); CLP+E-5 1.4mmol.l^−1^(1.2-1.7mmol.l^−1^), *p*=0.046)(Table 1). Compared to rats in the CLP group, Esmolol infused at 18mg.kg^-1^.h^-1^ reduced HR (CLP 371bpm(363-380bpm); CLP+E-18 245bpm(236-267bpm), *p*=0.001), but didn't worsen MAP (CLP77mmHg(71-86mmHg); CLP+E-18 92mmHg(80-104mmHg), *p*=0.529), and had no effects on circulating level of lactate (CLP 2.4mmol.l^−1^(2.1-3.2mmol.l^−1^); CLP+E-18 2.4mmol.l^−1^(2.1-2.6mmol.l^−1^), *p*=0.785)(Table 1). Infusion of Esmolol at 5mg.kg^-1^.h^-1^ improved CLP-induced 1) cardiac muscle fibers destruction, congestion and inflammatory infiltration (Supplemental Figure S4A, http://links.lww.com/SHK/B646); 2) inflammatory cells aggregation, intra-alveolar capillary hemorrhages and thickening of the alveolar walls in lung tissues (Supplemental Figure S4B, http://links.lww.com/SHK/B646); and 3) depletion of reticuloendothelial cells and lymphocytes in spleen tissues (supplemental Figure S4C, http://links.lww.com/SHK/B646). Compared to the CLP group, the heart (*p*=0.024), lung (*p*=0.012) and spleen (*p*=0.043) injury scores were reduced in the CLP+E-5 group (Table 1). Infusion of Esmolol at 18mg.kg^-1^.h^-1^ did not significantly improve CLP induced heart, lung and spleen injuries (heart: *p*=0.516; lung: *p*=0.117; spleen: *p*=0.086)(Table 1).

**18 hours-mortality**

Eighteen hours after CLP, the mortality rate was 0 of 8, 4 of 12, 1 of 9 and 2 of 10 respectively in Sham, CLP, CLP+E5 and CLP+E18 group (Supplemental Table S3, http://links.lww.com/SHK/B646).

**Effects of different doses of Esmolol on PBMCs apoptosis**

Compared to the sham group, CLP increased the apoptosis of monocytes (*p*=0.001), B-lymphocytes (*p*=0.001) and T-lymphocytes (*p*=0.001)(Figure 1). Infusion of Esmolol at both 5 and 18mg.kg^-1^.h^-1^ did not reduce CLP-induced apoptosis of monocytes (CLP; *vs.* CLP+E-5, *p*=0.993; *vs.* CLP+E-18, *p*=0.934)(Figure 1A) and B-lymphocytes (CLP; *vs.* CLP+E-5, *p*=0.989; *vs.* CLP+E-18, *p*=0.992)(Figure 1B). Infusion of Esmolol at 5mg.kg^-1^.h^-1^ significantly reduced apoptosis of T-lymphocytes (*p*=0.019)(Figure 1C). However, infusion of Esmolol at 18mg.kg^-1^.h^-1^ did not significantly reduced apoptosis of T-lymphocytes (*p*=0.999)(Figure 1C).

**Effects of different doses of Esmolol on** **Th0 differentiation**

CLP induced an increase in both Th1(*p*=0.005) and Th2 (*p*=0.001) levels compared to the sham group (Figure 2A and B). Infusion of Esmolol at both 5 and 18mg.kg^−1^.h^-1^ decreased CLP-induced increase of Th2 (CLP; *vs.* CLP+E-5, *p*=0.042; *vs.* CLP+E-18, *p*=0.029), but only at 18mg.kg^−1^.h^-1^ attenuated CLP-induced increase of Th1 (CLP; *vs.* CLP+E-5, *p*=0.232; *vs.* CLP+E-18, *p*=0.049). Thus, infusion of Esmolol at 5mg.kg^−1^.h^-1^ rather than 18mg.kg^−1^.h^-1^ lowered the ratio of Th2 to Th1 (CLP; *vs.* CLP+E-5, *p*=0.049; *vs.* CLP+E-18, *p*=0.992)(Figure 2C).

**Effects of different doses of Esmolol on** **apoptosis-associated signaling** **proteins**

Immunofluorescence analysis confirmed that β1-adrenoreceptors were expressed on the surface of splenic CD4^+^ T-lymphocytes (Supplemental Figure S5, http://links.lww.com/SHK/B646). Tested by western blot, CLP decreased the expression of p-Akt (*p*=0.010) and Bcl-2 (*p*=0.049) and increased cleaved Caspase-3 (*p*=0.001) compared to the Sham group (Figure 3A, B and C). Infusion of Esmolol at 5mg.kg^-1^.h^-1^ increased the expression of p-Akt (*p*=0.048) and Bcl-2 (*p*=0.032) and decreased cleaved Caspase-3 (*p*=0.048) compared to the CLP group (Figure 3A, B and C). However, infusion of Esmolol at 18mg.kg^-1^.h^-1^ did not increase expression of p-Akt (*p*=0.250) and Bcl-2 (*p*=0.256), which had a tendency to decrease cleaved Caspase-3 (*p*=0.054) compared to CLP group (Figure 3A, B and C). Then, immunohistochemistry confirmed the results of western blot (Sham *vs.* CLP: p-Akt, *p*=0.029; Bcl-2, *p*=0.029; cleaved Caspase-3, *p*=0.029; CLP *vs.* CLP+E-5: p-Akt, *p*=0.009; Bcl-2, *p*=0.033; cleaved Caspase-3, *p*=0.027; CLP *vs.*CLP+E-18: p-Akt, *p*=0.698; Bcl-2, *p*=0.628; cleaved Caspase-3, *p*=0.996) (Supplemental Figure S6A, B and C, http://links.lww.com/SHK/B646).

**Effects of different doses of Esmolol on Th0 differentiation-associated signaling proteins**

Compared to the sham group, CLP increased Erk1/2 phosphorylation analyzed by western blot (*p*=0.015) (Figure 3D)*.* Infusion of Esmolol at 5mg.kg^-1^.h^-1^ reduced CLP-induced increased in Erk1/2 phosphorylation (*p*=0.009)(Figure 3D). However, infusion of Esmolol at 18mg.kg^-1^.h^-1^ did not significantly decrease the CLP-induced increased in Erk1/2 phosphorylation (*p*=0.992)(Figure 4A). The results were confirmed by immunohistochemistry (CLP *vs.* sham, *p*=0.029; *vs.* CLP+E-5, *p*=0.041; *vs.* CLP+E-18, *p*=0.223)(Supplemental Figure S6D, http://links.lww.com/SHK/B646).

**Effect of different doses of esmolol on** **circulatory IL-4 level**

Compared to the sham group, CLP was associated with increased plasma levels of IL-4 (*p*＜0.001)(Supplemental Figure S7, http://links.lww.com/SHK/B646). Addition of esmolol at 5mg.kg^-1^.h^-1^ in CLP rats resulted in a decrease in plasma IL-4 level (*p*=0.031). However, there were no significant difference in plasma IL-4 level between the CLP and CLP+E-18 groups (*p*=0.837).

**Effects of different doses of Esmolol on the β1-adrenoreceptor expression on T- lymphocytes**

Compared to the sham group, CLP decreased mRNA (*p*=0.002) and protein (*p*=0.029) expression of β1-adrenoreceptor in splenic CD4^+^ T-lymphocytes tested by qRT-PCR and immunohistochemistry (Figure 4 and Supplemental Figure S8, http://links.lww.com/SHK/B646). Infusion of Esmolol at both 5 and 18mg.kg^-1^.h^-1^ didn't modulate the mRNA (CLP *vs.* CLP+E-5, *p*=0.669; *vs.* CLP+E-18, *p*=0.869)(Figure 4A) and protein (CLP *vs.* CLP+E-5, *p*=0.352; *vs.* CLP+E-18, *p*=0.824)(Figure 4B) expression of β1-adrenoreceptor in splenic CD4^+^ T lymphocytes.

**Discussion**

The main result of the study is that blocking β1-adrenoreceptors by Esmolol decreased circulating T-lymphocyte apoptosis and restored peripheral blood Th2/Th1 ratio in septic shock model. Akt/Bcl-2/Caspase-3 pathway, which was associated with T-lymphocyte apoptosis, was found to be modulated by Esmolol. Erk1/2 activity, which promoted Th0 differentiation to Th2, was revealed to be inhibited by Esmolol. Esmolol at low dose without heart rate reduction showed better immunomodulatory effects than at high dose with heart rate reduction.

**Model characteristics**

In the study, the CLP model was used to establish the septic shock model. All rats were resuscitated with adapted fluids. Antibiotics was infused 4 hours after CLP to mimic clinical settings. As in previous studies(9, 10, 20, 23), all rats that underwent CLP showed the typical characteristics of septic shock, including hypotension, hyperlactatemia and multiple organ injuries, including heart, lung and spleen (Table 1 and Supplemental Figure S4, http://links.lww.com/SHK/B646).

**Effects of Esmolol on survival**

Ackland *et al.*(24) pre-treated sepsis rats with β1-adrenoreceptor blockers (metoprolol and atenolol) two days before injection of lipopolysaccharide (LPS) improved survival. Metoprolol increased median time to death in sepsis rats when pre-treated two days before CLP. However, both metoprolol and atenolol failed to improve survival when treatment commenced 6 hours after induction of sepsis in their study. The doses used of metoprolol and atenolol resulted in a 20% reduction in the heart rate from baseline in the study. Medical treatment is usually after sepsis insult in clinical settings. To reproduce clinical setting of septic patients, the following studies commenced treatment after sepsis insult. Mori *et al.* (25) administrated septic rats with Esmolol infusion one hour after CLP. The dose of Esmolol also reduced heart rate by approximately 20% as compared with baseline. They found the survival time was significantly improved in Esmolol group. Kimmoun *et al.*(9) infused septic rats with Esmolol four hours after CLP with a dose reducing heart rate as compared to CLP group. Median time to death was also increased in Esmolol-treated septic rats. In contrast to previous studies, Ibrahim-zada *et al.* (26) infused sepsis rats with Esmolol four hours after injection of LPS with a very low dose without any effect on myocardial function and also showed survival improvement in Esmolol group. Previous results showed that both high and low doses of β1-adrenoreceptor blockers improved survival in experimental sepsis. In our study, the 18 hours-mortality was 33.3%, 11.1% and 20% respectively in CLP, CLP+E-5 and CLP+18 group, which was consistent with previous studies(9, 24-26).

**Effects of Esmolol on T-lymphocyte apoptosis**

Previous studies have reported that immunosuppression predominately results from apoptosis of monocytes, B-lymphocytes and T-lymphocytes in septic shock patients(3, 27). In our study, CLP induced apoptosis of circulating monocytes, B-lymphocytes and T-lymphocytes, which was similar to the clinical settings. Overstimulation of immune cells via adrenergic receptors by catecholamines, which are secreted by the sympathetic nervous system over-activation contributes to their apoptosis in septic shock(28, 29). Our results showed that blocking β1-adrenergic receptors by Esmolol at low dose significantly reduced T-lymphocyte apoptosis in rats with septic shock.

**Mechanisms of Esmolol on T-lymphocyte apoptosis**

β1-adrenoreceptor could modulate Akt via Gβγ/PI3K(13). Our previous study showed that blocking β1-adrenoreceptors by Esmolol could increase Akt phosphorylation(10). Akt activation leads to Bad phosphorylation resulting in Bcl-2 release, ultimately promoting cell survival(14). Our results revealed that low dose of Esmolol increased Akt phosphorylation and Bcl-2 expression and reduced cleaved Caspase-3 in splenic CD4^+^ T-lymphocytes in septic shock models. The results were consistent with Qi *et al's* report in cardiomyocytes that phosphorylation of Akt induced up-regulation of Bcl-2 and down-regulation of cleaved Caspase-3(15). Therefore, Esmolol might reduce T-lymphocytes apoptosis in septic shock by modulating the Akt/Bcl-2/Caspase-3 pathway (Figure 5).

**Effects of Esmolol on Th2/Th1 ratio**

Previous studies have shown an imbalance of T-lymphocyte sub-populations in septic patients, such as an augmented Th2/Th1 ratio(5, 30, 31). Our results showed an increased peripheral blood Th2/Th1 ratio in septic shock models as clinical settings. Infusion of Esmolol at low dose restored the ratio of Th2 and Th1 in septic shock.

**Mechanisms of Esmolol on Th2 differentiation**

Induction of Th0 into the Th2 differentiation pathway depends to a significant extent on IL-4 produced upon TCR cross-linking(32)*.* IL-4 establishes a positive feedback loop through IL-4R, which further reinforces IL4 expression while silencing the IFN-γ locus at the same time in Th0(18, 19). Erk influences TCR-dependent activation of IL4 gene transcription through association to the proximal promoter. Thus, Erk modulates Th0 differentiation via TCR-dependent IL-4 production. Activation of the β1-adrenoreceptor could induce Erk phosphorylation through the AC-cAMP-PKA pathway(16). Our results showed that blocking β1-adrenoreceptor by Esmolol decreased CLP-induced Erk1/2 phosphorylation. Circulating IL-4 level was also decreased in Esmolol-treated group, which was consistent with Manon *et al.*'s study(11). In their study, blocking β1-adrenoreceptor by Esmolol decreased circulating IL-4 level in septic shock mice. These findings supported that Esmolol might reduce Th0 differentiation to Th2 in septic shock by decreasing IL-4 production via inhibiting of Erk1/2 activation (Figure 5).

**Different doses of Esmolol on immunomodulation**

The optimal dose of Esmolol for immunoregulation of septic shock remains unclear. Our results showed that Esmolol at low dose without heart rate reduction rather than at high dose with heart rate reduction significantly decreased T-lymphocytes apoptosis and restored Th2/Th1 ratio in septic shock models. Thus, low dose of Esmolol might be more promising for modulating the immune response in septic shock. More studies are needed to confirm our findings.

**Effects of Esmolol on β1-adrenoreceptor on T-lymphocytes**

In septic shock, excessive stimulation by catecholamine resulted in the reduction of β1-adrenoreceptor density in cardiomyocytes, which was restored by Emolol treatment(6). Our results demonstrated that the mRNA and protein expression of β1-adrenoreceptor also decreased in T-lymphocytes in septic shock rats. However, Esmolol infusion at different doses did not restore the mRNA or protein expression of β1-adrenoreceptor on T-lymphocytes in septic shock rats. The effects of Esmolol on its receptor expression may vary in different cells. Further study is needed to elucidate the mechanisms.

**Study limitation**

In fact, Esmolol also decreased peripheral blood Th0 differentiation to Th1 in septic shock in the study. However, the mechanisms were not explored in the study. Our work is just a starting point for investigating the effects and mechanisms of Esmolol on Th0 differentiation. More Follow-up work is needed. Besides, the immune status varies during the course of septic shock. The optimal time to the initiation of Esmolol treatment requires future investigation. Lastly, only male rats were used in this study. Female rats should be used in the following researches to complete date for the entire population.

**Conclusions**

Esmolol reduced circulating T-lymphocyte apoptosis and restored the peripheral blood Th2/Th1 ratio. Esmolol might modulate the Akt/Bcl-2/Caspase-3 pathway to relieve T-lymphocyte apoptosis and inhibit Erk1/2 activity to decrease peripheral blood Th0 differentiation to Th2. Esmolol at low dose without heart rate reduction showed better immunomodulatory effects than at high dose. Esmolol at low dose may be a potential immunoregulator of septic shock.

**Supplementary Information**

Supplemental Tables and Figures are presented in the Supplementary Material.

**Acknowledgments**

We thank the department of anatomy and embryology in Wuhan University for their technical support.

**Conflicts of interest**

All authors have disclosed that they do not have any potential conflicts of interest.

**Authors' contributions**

Drs. Wei and Yang take responsibility for content of the manuscripts. Drs. Ma, Cheng, and Zheng contribute equally to the study and share the first authorship. Drs. Wei and Yang contribute equally to the study. Drs. Wei and Ma contributed to the study concept and design. Drs. Ma, Zheng, He, and Zhou were involved in experiments. Drs. Ma and Cheng contributed to the statistical analysis. Drs. Wei, Yang, Ma, Wang, and Cheng contributed to the drafting of the article. All authors read and approved the final manuscript.

**References**

1. M. Singer, C. S. Deutschman, C. W. Seymour, M. Shankar-Hari, D. Annane, M. Bauer, R. Bellomo, G. R. Bernard, J. D. Chiche, C. M. Coopersmith, R. S. Hotchkiss, M. M. Levy, J. C. Marshall, G. S. Martin, S. M. Opal, G. D. Rubenfeld, T. van der Poll, J. L. Vincent and D. C. Angus: The Third International Consensus Definitions for Sepsis and Septic Shock (Sepsis-3). *JAMA* 315(8):801-10, 2016.
2. R. S. Hotchkiss, G. Monneret and D. Payen: Sepsis-induced immunosuppression: from cellular dysfunctions to immunotherapy. *Nat Rev Immunol* 13(12):862-74, 2013.
3. M. J. Delano and P. A. Ward: The immune system's role in sepsis progression, resolution, and long-term outcome. *Immunol Rev* 274(1):330-353, 2016.
4. J. S. Boomer, K. To, K. C. Chang, O. Takasu, D. F. Osborne, A. H. Walton, T. L. Bricker, S. D. Jarman, 2nd, D. Kreisel, A. S. Krupnick, A. Srivastava, P. E. Swanson, J. M. Green and R. S. Hotchkiss: Immunosuppression in patients who die of sepsis and multiple organ failure. *JAMA* 306(23):2594-605, 2011.
5. N. R. Ferguson, H. F. Galley and N. R. Webster: T helper cell subset ratios in patients with severe sepsis. *Intensive Care Med* 25(1):106-9, 1999.
6. T. Suzuki, H. Morisaki, R. Serita, M. Yamamoto, Y. Kotake, A. Ishizaka and J. Takeda: Infusion of the beta-adrenergic blocker esmolol attenuates myocardial dysfunction in septic rats. *Crit Care Med* 33(10):2294-301, 2005.
7. A. Morelli, C. Ertmer, M. Westphal, S. Rehberg, T. Kampmeier, S. Ligges, A. Orecchioni, A. D'Egidio, F. D'Ippoliti, C. Raffone, M. Venditti, F. Guarracino, M. Girardis, L. Tritapepe, P. Pietropaoli, A. Mebazaa and M. Singer: Effect of heart rate control with esmolol on hemodynamic and clinical outcomes in patients with septic shock: a randomized clinical trial. *JAMA* 310(16):1683-91, 2013.
8. A. Morelli, M. Singer, V. M. Ranieri, A. D'Egidio, L. Mascia, A. Orecchioni, F. Piscioneri, F. Guarracino, E. Greco, M. Peruzzi, G. Biondi-Zoccai, G. Frati and S. M. Romano: Heart rate reduction with esmolol is associated with improved arterial elastance in patients with septic shock: a prospective observational study. *Intensive Care Med* 42(10):1528-1534, 2016.
9. A. Kimmoun, H. Louis, N. Al Kattani, J. Delemazure, N. Dessales, C. Wei, P. Y. Marie, K. Issa and B. Levy: beta1-Adrenergic Inhibition Improves Cardiac and Vascular Function in Experimental Septic Shock. *Crit Care Med* 43(9):e332-40, 2015.
10. C. Wei, H. Louis, M. Schmitt, E. Albuisson, S. Orlowski, B. Levy and A. Kimmoun: Effects of low doses of esmolol on cardiac and vascular function in experimental septic shock. *Crit Care* 20(1):407, 2016.
11. M. Durand, E. Hagimont, H. Louis, P. Asfar, J. P. Frippiat, M. Singer, G. Gauchotte, C. Labat, P. Lacolley, B. Levy, B. Glenn Chousterman and A. Kimmoun: The beta1-Adrenergic Receptor Contributes to Sepsis-Induced Immunosuppression Through Modulation of Regulatory T-Cell Inhibitory Function. *Crit Care Med*, 2022.
12. D. K. Vassilatis, J. G. Hohmann, H. Zeng, F. Li, J. E. Ranchalis, M. T. Mortrud, A. Brown, S. S. Rodriguez, J. R. Weller, A. C. Wright, J. E. Bergmann and G. A. Gaitanaris: The G protein-coupled receptor repertoires of human and mouse. *Proc Natl Acad Sci U S A* 100(8):4903-8, 2003.
13. L. Sun and R. D. Ye: Role of G protein-coupled receptors in inflammation. *Acta Pharmacologica Sinica* 33(3):342-350, 2012.
14. H. U. A. Zhang, Z. Xiong, J. Wang, S. Zhang, L. E. I. Lei, L. I. Yang and Z. Zhang: Glucagon-like peptide-1 protects cardiomyocytes from advanced oxidation protein product-induced apoptosis via the PI3K/Akt/Bad signaling pathway. *Molecular Medicine Reports* 13(2):1593-1601, 2016.
15. Z. Qi, R. Wang, R. Liao, S. Xue and Y. Wang: Neferine Ameliorates Sepsis-Induced Myocardial Dysfunction Through Anti-Apoptotic and Antioxidative Effects by Regulating the PI3K/AKT/mTOR Signaling Pathway. *Front Pharmacol* 12:706251, 2021.
16. J. Zheng, H. Shen, Y. Xiong, X. Yang and J. He: The beta1-adrenergic receptor mediates extracellular signal-regulated kinase activation via Galphas. *Amino Acids* 38(1):75-84, 2010.
17. P. Tripathi, N. Sahoo, U. Ullah, H. Kallionpaa, A. Suneja, R. Lahesmaa and K. V. Rao: A novel mechanism for ERK-dependent regulation of IL4 transcription during human Th2-cell differentiation. *Immunol Cell Biol* 90(7):676-87, 2012.
18. K. M. Ansel, I. Djuretic, B. Tanasa and A. Rao: Regulation of Th2 differentiation and Il4 locus accessibility. *Annu Rev Immunol* 24:607-56, 2006.
19. K. M. Murphy and S. L. Reiner: The lineage decisions of helper T cells. *Nat Rev Immunol* 2(12):933-44, 2002.
20. C. Wei, N. Al Kattani, H. Louis, E. Albuisson, B. Levy and A. Kimmoun: If Channel Inhibition With Ivabradine Does Not Improve Cardiac and Vascular Function in Experimental Septic Shock. *Shock* 46(3):297-303, 2016.
21. A. E. Khodir, Y. A. Samra and E. Said: A novel role of nifuroxazide in attenuation of sepsis-associated acute lung and myocardial injuries; role of TLR4/NLPR3/IL-1beta signaling interruption. *Life Sci* 256:117907, 2020.
22. D. R. Principe, A. M. Diaz, C. Torres, R. J. Mangan, B. DeCant, R. McKinney, M. S. Tsao, A. Lowy, H. G. Munshi, B. Jung and P. J. Grippo: TGFbeta engages MEK/ERK to differentially regulate benign and malignant pancreas cell function. *Oncogene* 36(30):4336-4348, 2017.
23. C. Cao, M. Yu and Y. Chai: Pathological alteration and therapeutic implications of sepsis-induced immune cell apoptosis. *Cell Death Dis* 10(10):782, 2019.
24. G. L. Ackland, S. T. Yao, A. Rudiger, A. Dyson, R. Stidwill, D. Poputnikov, M. Singer and A. V. Gourine: Cardioprotection, attenuated systemic inflammation, and survival benefit of beta1-adrenoceptor blockade in severe sepsis in rats. *Crit Care Med* 38(2):388-94, 2010.
25. K. Mori, H. Morisaki, S. Yajima, T. Suzuki, A. Ishikawa, N. Nakamura, Y. Innami and J. Takeda: Beta-1 blocker improves survival of septic rats through preservation of gut barrier function. *Intensive Care Med* 37(11):1849-56, 2011.
26. I. Ibrahim-Zada, P. Rhee, C. T. Gomez, J. Weller and R. S. Friese: Inhibition of sepsis-induced inflammatory response by beta1-adrenergic antagonists. *J Trauma Acute Care Surg* 76(2):320-7; discussion 327-8, 2014.
27. R. Shao, Y. Fang, H. Yu, L. Zhao, Z. Jiang and C. S. Li: Monocyte programmed death ligand-1 expression after 3-4 days of sepsis is associated with risk stratification and mortality in septic patients: a prospective cohort study. *Crit Care* 20(1):124, 2016.
28. R. F. Stolk, M. Kox and P. Pickkers: Noradrenaline drives immunosuppression in sepsis: clinical consequences. *Intensive Care Med* 46(6):1246-1248, 2020.
29. J. L. Jiang, Y. P. Peng, Y. H. Qiu and J. J. Wang: Adrenoreceptor-coupled signal-transduction mechanisms mediating lymphocyte apoptosis induced by endogenous catecholamines. *J Neuroimmunol* 213(1-2):100-11, 2009.
30. M. Xue, J. Xie, L. Liu, Y. Huang, F. Guo, J. Xu, Y. Yang and H. Qiu: Early and dynamic alterations of Th2/Th1 in previously immunocompetent patients with community-acquired severe sepsis: a prospective observational study. *J Transl Med* 17(1):57, 2019.
31. M. Xue, Y. Tang, X. Liu, M. Gu, J. Xie, L. Liu, Y. Huang, F. Guo, Y. Yang and H. Qiu: Circulating Th1 and Th2 Subset Accumulation Kinetics in Septic Patients with Distinct Infection Sites: Pulmonary versus Nonpulmonary. *Mediators Inflamm* 2020:8032806, 2020.
32. N. Noben-Trauth, J. Hu-Li and W. E. Paul: Conventional, naive CD4+ T cells provide an initial source of IL-4 during Th2 differentiation. *J Immunol* 165(7):3620-5, 2000.

Table

Table 1 Comparison of hemodynamics and organ injury scores 18 hours after operation in different groups

**Figure legends**

**Figure 1 Effects of different doses of Esmolol on apoptosis of PBMCs by flow cytometry** Apoptosis rates of circulating monocytes (A), B-lymphocytes (B) and T-lymphocytes (C) in different groups were shown in the histogram. Data are expressed as median ± interquartile range, n=8. The upper edges of error bars represent the 75th percentile in each group. **p* < 0.05: CLP group *vs.* Sham group; #*p* < 0.05: CLP+E-5 *vs.* CLP group. CLP: cecal ligation and puncture; CLP+E-5: CLP with Esmolol infused at 5 mg.kg^−1^.h^−1^; CLP+E-18: CLP with Esmolol infused at 18mg.kg^−1^.h^−1^.

**Figure 2 Effects of different doses of Esmolol on T-lymphocyte subsets by flow cytometry** The percentages of Th1 (A) and Th2 (B) in T help cells (Th) and the ratio of Th2/Th1 (C) were shown in the histogram. Data are expressed as median ± interquartile range, n=8. The upper edges of error bars represent the 75th percentile in each group. * *p* < 0.05: CLP group *vs.* Sham group; # *p* < 0.05: CLP+E-5,CLP+E-18 *vs.* CLP group. CLP: cecal ligation and puncture; CLP+E-5: CLP with Esmolol infused at 5 mg.kg^−1^.h^−1^; CLP+E-18: CLP with Esmolol infused at 18mg.kg^−1^.h^−1^.

**Figure 3 Effects of different doses of Esmolol on apoptosis-associated signaling proteins by western blots** Western blots revealed phosphorylated Akt (p-Akt) (A), Bcl-2 (B), cleaved Caspase-3 (C) and phosphorylated Erk1/2 (p-Erk1/2) (D). Proteins were obtained from splenic CD4^+^ T-lymphocytes lysates (n=8) prepared from all experimental rat groups. Two typical western blots are shown below each histogram. Densitometric analysis was used to calculate the normalized protein ratio. Data are expressed as median ± interquartile range. The upper edges of error bars represent the 75th percentile in each group. * *p* < 0.05: CLP group *vs.* Sham group; # *p* < 0.05: CLP+E-5 *vs.* CLP group. CLP: cecal ligation and puncture; CLP+E-5: CLP with Esmolol infused at 5 mg.kg^−1^.h^−1^; CLP+E-18: CLP with Esmolol infused at 18mg.kg^−1^.h^−1^.

**Figure 4 Effects of Esmolol on mRNA and protein expression of β1-adrenoceptors on Splenic CD4^+^ T-lymphocytes** mRNA expression levels of β1-adrenoceptor (A) were evaluated by real-time PCR (qRT-PCR) (n=6). The results were first normalized to one housekeeping gene and thereafter to sham expression, which was set at 1. Immunohistochemistry revealed β1-adrenoceptor in the splenic tissues. The staining intensity was used to evaluate β1-adrenoceptor (B) expression level (n=4). Data are expressed as median ± interquartile range. The upper edges of error bars represent the 75th percentile in each group. **p* < 0.05: CLP group *vs.* Sham group; CLP: cecal ligation and puncture; CLP+E-5: CLP with Esmolol infused at 5 mg.kg^−1^.h^−1^; CLP+E-18: CLP with Esmolol infused

at 18mg.kg^−1^.h^−1^.

**Figure 5 Signaling pathways of β1-adrenoreceptors involved in inflammation** Agonist binding stimulates β1-adrenoreceptors (β1-AR) and results in coupling with and activation of G protein, which dissociates into Gs and Gβγ subunits. The Gβγ inhibits Akt phosphorylation, which subsequently decrease Bcl-2 release resulting in Caspase-3 cleaved, finaly promotes cell apoptosis. The Gs via several downstream signaling factors induces activation of Erks transcription factors, which induce IL-4 production promoting Th2 differentiation. The red arrows show the effects of blocking β1-AR by Esmolol on its downstream signaling pathway.
