# Supplementary material for: LOW DOSE OF ESMOLOL ATTENUATES SEPSIS-INDUCED IMMUNOSUPPRESSION VIA MODULATING T-LYMPHOCYTE APOPTOSIS AND DIFFERENTIATION
Source: Shock. 2023 Feb 28;59(5):771–8. doi: 10.1097/SHK.0000000000002104 (PMC10125111; doi:10.1097/SHK.0000000000002104)
Supplement: SUPPLEMENTARY MATERIAL [file shock-59-771-s002.docx]

**Low dose of Esmolol attenuates sepsis-induced immunosuppression via modulating T-lymphocyte apoptosis and differentiation**

Ying Ma ^1,2^, Zhenshun Cheng ^1,2,4^, Yong Zheng ^3^, Wei Wang ^1^, Shaojun He ^1^, Xiaolian Zhou ^1^, Jiong Yang ^1^, Chaojie Wei ^1,2^

1. Department of Pulmonary and Critical Care Medicine, Zhongnan Hospital of Wuhan University, Wuhan, 430071, Hubei, China
2. Wuhan Research Center for Infectious Diseases and Cancer, Chinese Academy of Medical Sciences, Wuhan, 430071, Hubei, China
3. Department of Anatomy and Embryology, Wuhan University Taikang Medical School (School of Basic Medical Sciences), Wuhan, 430071, Hubei, China
4. Hubei Engineering Center for Infectious Disease Prevention, Control and Treatment, Wuhan, 430071, Hubei, China

**Corresponding author information ：**

Corresponding author 1: Chaojie Wei, Department of Pulmonary and Critical Care Medicine, Zhongnan Hospital of Wuhan University, Wuhan, 430071, China. E-mail: chaojie_wei@163.com

Corresponding author 2: Jiong Yang, Department of Pulmonary and Critical Care Medicine, Zhongnan Hospital of Wuhan University, Wuhan, 430071, China. E-mail: yangjiongwh@126.com

***Supplemental Digital Content***

[Supplemental tables 3](#_Toc9004)

[Table S1 Antibodies' information](#_Toc14656) 3

[Table S2 The primer sequences for qRT-PCR 4](#_Toc13151)

[Table S3 The outcome of operated rats 5](#_Toc13151)

[Supplemental figures](#_Toc9004) 6

[Figure S1 Gating strategy for flow cytometric analysis of circulating monocytes, B-lymphocytes and T-lymphocytes](#_Toc13555) 6

[Figure S2 Gating strategy for flow cytometric analysis of circulating T-lymphocyte subsets 7](#_Toc13555)

[Figure S3 Detection of the purity of CD4](#_Toc10499)^[+](#_Toc10499)^ [T-lymphocytes in rat spleens 8](#_Toc10499)

[Figure S4 Effects of different doses of Esmolol on CLP-induced heart, lung and spleen injuries](#_Toc10711) 9

[Figure S5 Confirmation of expression of β1-adrenoreceptor on splenetic CD4](#_Toc17603)^[+](#_Toc17603)^ [T-lymphocytes](#_Toc17603) 10

[Figure S6 Effects of different doses of Esmolol on apoptosis-associated signaling proteins and naive CD4](#_Toc17603)^[+](#_Toc17603)^ [T cells (Th0) differentiation-associated signaling protein by immunohistochemistry](#_Toc17603) 11

[Figure S7 Effects of different doses of Esmolol](#_Toc15990) [on circulatory IL-4 level 1](#_Toc15990)2

[Figure S8 Effects of Esmolol on protein expression of β1-adrenoceptors on Splenic CD4](#_Toc15990)^[+](#_Toc15990)^ [T-lymphocytes by immunohistochemistry 1](#_Toc15990)3

# Supplemental tables

Table S1 Antibodies' information

| **Method** | **Antibody name** | **Dilution** | **Providers** |
| --- | --- | --- | --- |
| Flow cytometry | PE-labeled anti-CD11a | - | BD Pharmingen™, USA |
|  | APC-labeled anti-CD11b | - | BD Pharmingen™, USA |
|  | APC/Cy7-labeled anti-CD45RA | - | BD Pharmingen™, USA |
|  | BV421-labeled anti-CD3 | - | BD Pharmingen™, USA |
|  | FITC Annexin V Apoptosis Detection Kit with 7-AAD | - | Biolegend, USA |
|  | PE/Cy7-labeled anti-CD4 | - | BD Pharmingen™, USA |
|  | APC-labeled anti-INF-γ | - | BD Pharmingen™, USA |
|  | PE-labeled anti-IL-4 | - | BD Pharmingen™, USA |
| Isolation of splenic CD4^+^ T-lymphocytes | PE/Cy7-labeled anti-CD4 | - | BD Pharmingen™, USA |
| Western blotting | anti-p-Akt | 1:1000 | Cell Signaling Technology, USA |
|  | anti-Akt | 1:1000 | Cell Signaling Technology, USA |
|  | anti-p-Erk1/2 | 1:1000 | Cell Signaling Technology, USA |
|  | anti-Erk1/2 | 1:1000 | Cell Signaling Technology, USA |
|  | anti-Bcl-2 | 1:500 | Abcam, USA |
|  | anti-Cleaved-Caspase-3 | 1:1000 | Cell Signaling Technology, USA |
|  | anti-GAPDH | 1:10000 | Abcam, USA |
|  | HRP-conjugated  secondary antibody | 1:10000 | Aspen, China |
| Immunohistochemistry | p-Akt | 1:400 | Affinity, USA |
|  | Bcl-2 | 1:100 | BOSTER, China |
|  | Cleaved Caspase-3 | 1:100 | Cell Signaling Technology, USA |
|  | p-Erk1/2 | 1:100 | Cell Signaling Technology, USA |
|  | anti-β1 antibody | 1:800 | ABclonal, China |
|  | HRP-conjugated  secondary antibody | 1:500 | Abbkine, China |
| Immunofluorescence | anti-β1AR antibody | 1:50 | ABclonal, China |
|  | anti-CD4 antibody | 1:200 | ABclonal, China |

Table S2 The primer sequences for qRT-PCR

| **Gene** | **Forward Primer Sequence (5’-3')** | **Reverse Primer Sequence (3’-5')** |
| --- | --- | --- |
| β1-adrenoreceptor | CCAACCTCTTCATCA | CACATAGCACGTCT |

qRT-PCR: quantitative real-time PCR

Table S3 The outcome of operated rats

| **Group** | **Total** | **Died** | **Mortality(%)** |
| --- | --- | --- | --- |
| Sham | 8 | 0 | 0 |
| CLP | 12 | 4 | 33.3 |
| CLP+E-5 | 9 | 1 | 11.1 |
| CLP+E-18 | 10 | 2 | 20 |

# Supplemental figures


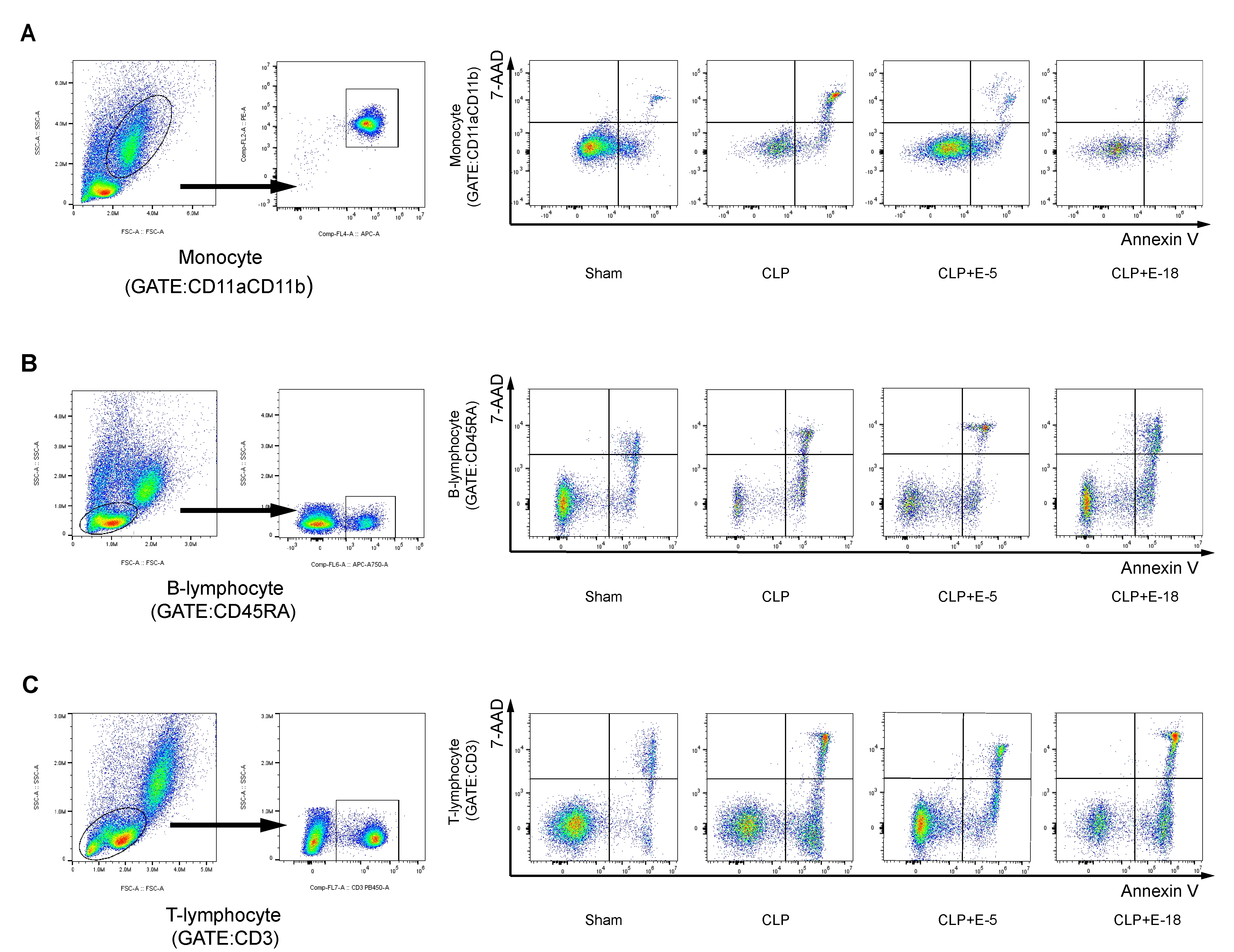


Figure S1 Gating strategy for flow cytometric analysis of circulating monocytes, B-lymphocytes and T-lymphocytes. Peripheral blood mononuclear cells (PBMCs) were stained with PE-labeled anti-CD11a, APC-labeled anti-CD11b antibodies, APC/Cy7-labeled anti-CD45RA and BV421-labeled anti-CD3 antibodies. (A) Monocytes were defined as CD11a^+^CD11b^+^ cells. (B) B-lymphocytes were defined as CD45RA^+^ cells. (C) T-lymphocytes were defined as CD3^+^ cells. Density plots of monocytes, B-lymphocytes and T-lymphocytes apoptosis for one representative rat per group are shown in the panel.


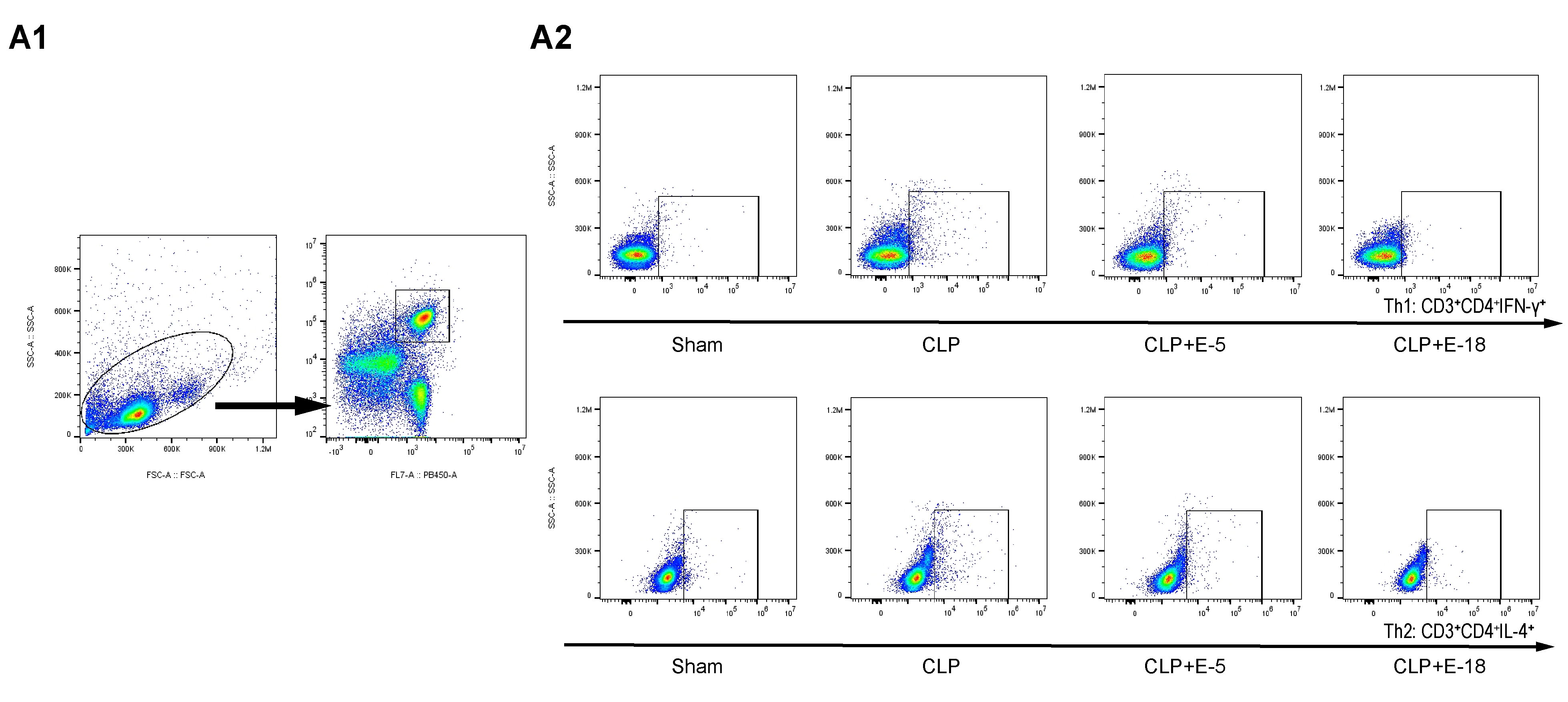


Figure S2 Gating strategy for flow cytometric analysis of circulating T-lymphocyte subsets CD3^+^CD4^+^ cells were analyzed and further specified T helper cells (A1). Th1 cells are defined as INF-γ^+^ cells in CD3^+^CD4^+^cells. Th2 cells are defined IL-4^+^ cells in CD3^+^CD4^+^ cells. Density plots of T helper 1 cells (Th1) and T helper 2 cells (Th2) for one representative rat per group are shown in panel (A2).


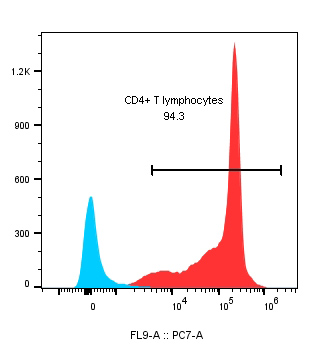


Figure S3 Detection of the purity of CD4^+^ T-lymphocytes in rat spleens. Representative flow cytometrical plot of the splenic CD4^+^ T-lymphocytes after magnetic cell separation. The blue histogram showed the splenic CD4^+^ T-lymphocytes without PE/CY7-labeled anti-CD4 antibody staining. The red histogram showed the splenic CD4^+^ T cells stained by PE/CY7-labeled anti-CD4 antibody.


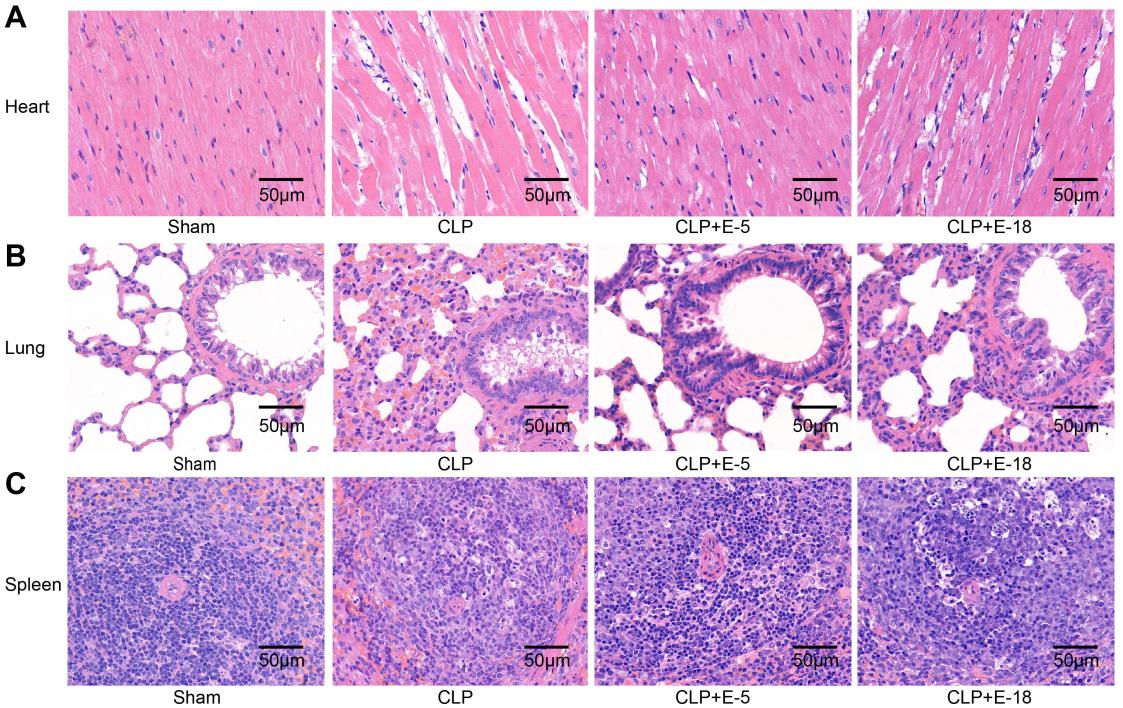


**Figure S4 Effects of different doses of Esmolol on CLP-induced heart, lung and spleen injuries.** Heart (A), lung (B) and spleen (C) tissue were stained with hematoxylin-eosin (H&E). The representative sections are shown at×200 original magnification and scale bars are 50 mm.


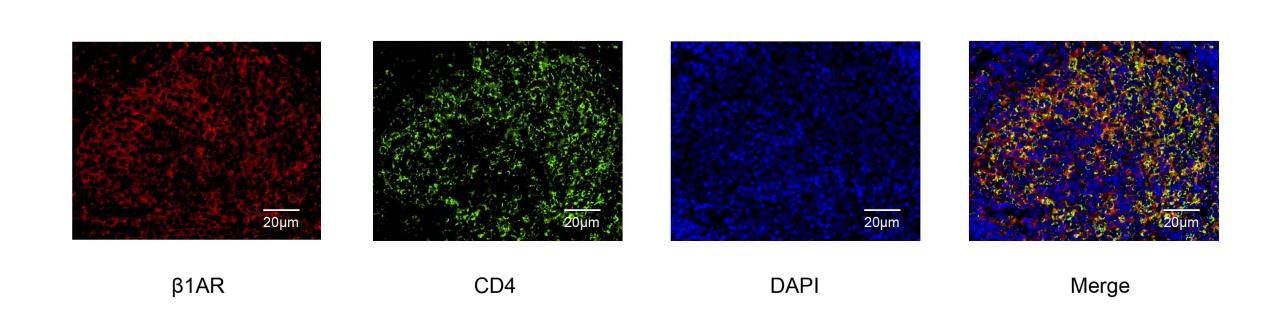


Figure S5 Confirmation of expression of β1-adrenoreceptor on splenetic CD4+ T-lymphocytes. Immunofluorescence of β1-adrenoreceptor and CD4 in the healthy rat spleen tissue. Three-color staining for anti-β1-adrenoreceptor antibody (red), anti-CD4 (green), and nucleus (blue). The representative sections are shown at×400 original magnification, and scale bars are 20 mm.

**
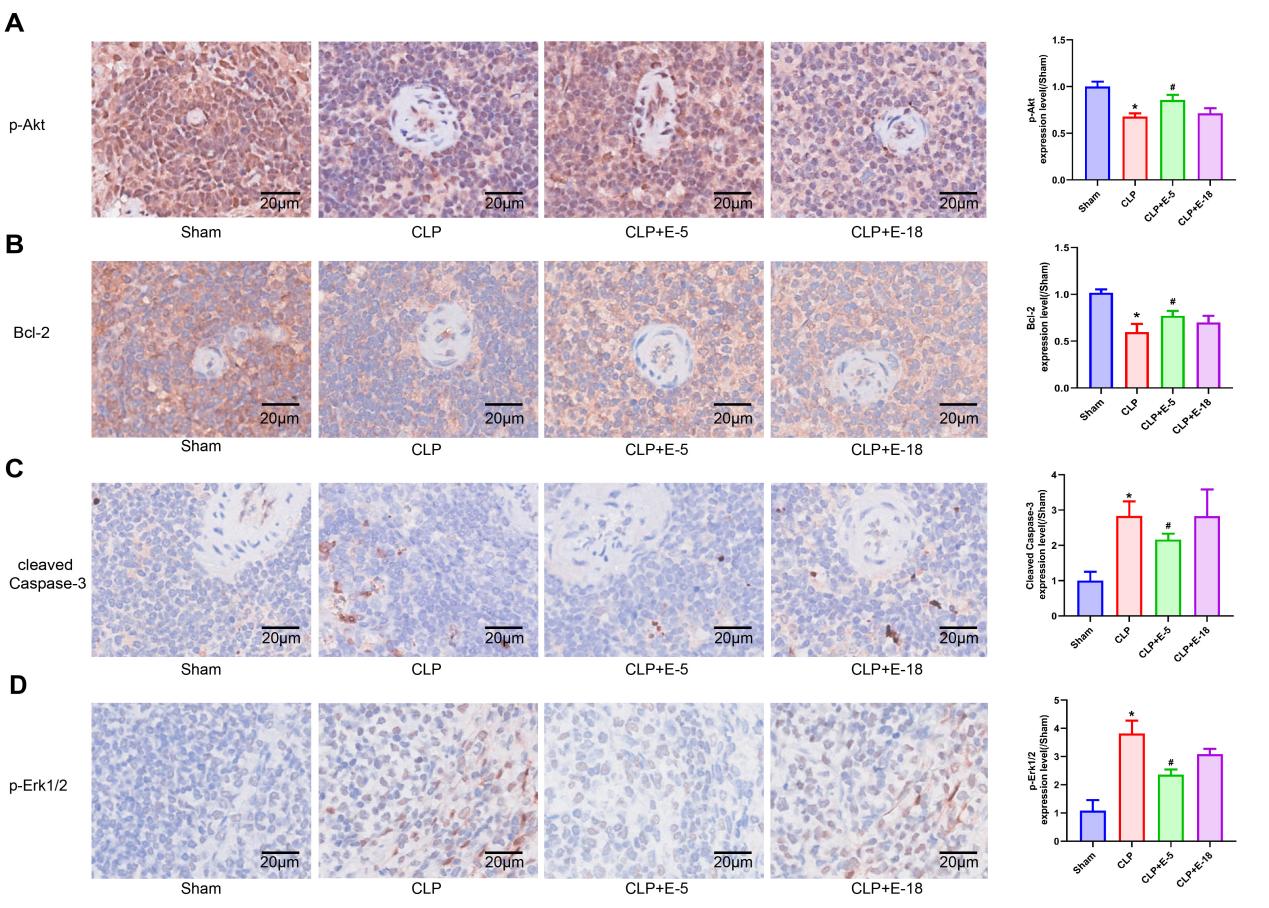
**

Figure S6 Effects of different doses of Esmolol on apoptosis-associated signaling proteins and naive CD4^+^ T cells (Th0) differentiation-associated signaling protein by immunohistochemistry. Immunohistochemistry revealed phosphorylated Akt (p-Akt), Bcl-2, cleaved Caspase-3 and phosphorylated Erk1/2 (p-Erk1/2) in the splenic tissues. Immunohistochemistry's staining intensity (n=4) was used to evaluate p-Akt (A), Bcl-2 (B), cleaved Caspase-3 (C) and p-Erk1/2 (D) expression levels. The representative sections are shown at×400 original magnification, and scale bars are 20 mm. Data are expressed as median ± interquartile range. The upper edges of error bars represent the 75th percentile in each group. **p* < 0.05: CLP group *vs.* Sham group; #*p* < 0.05: CLP+E-5 *vs.* CLP group. CLP: cecal ligation and puncture; CLP+E-5: CLP with Esmolol infused at 5 mg.kg^−1^.h^−1^; CLP+E-18: CLP with Esmolol infused at 18mg.kg^−1^.h^−1^.


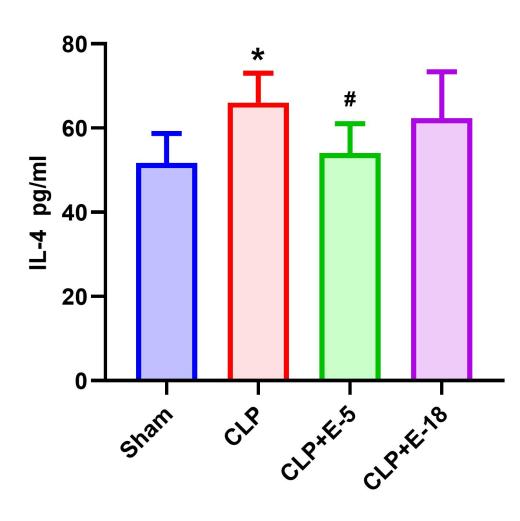


Figure S7 Effects of different doses of Esmolol on on circulatory IL-4 level. Assessment of circulatory cytokine level of IL-4 as measured by ELISA(n=8). Data are expressed as median ± interquartile range. The upper edges of error bars represent the 75th percentile in each group. **p* < 0.05: CLP group *vs.* Sham group; #*p* < 0.05: CLP+E-5 *vs.* CLP group. CLP: cecal ligation and puncture; CLP+E-5: CLP with Esmolol infused at 5 mg.kg^−1^.h^−1^; CLP+E-18: CLP with Esmolol infused at 18mg.kg^−1^.h^−1^.


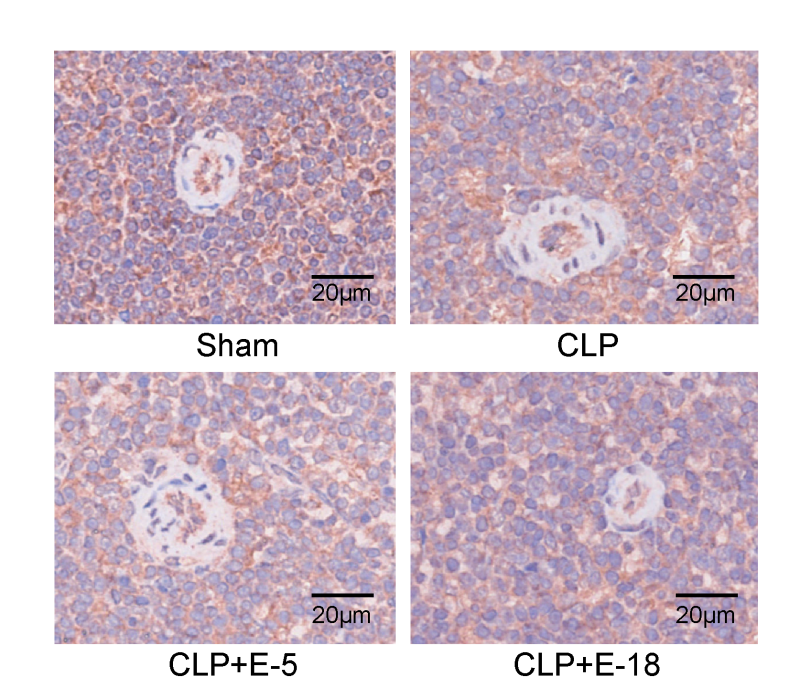


Figure S8 Effects of Esmolol on protein expression of β1-adrenoceptors on Splenic CD4^+^ T-lymphocytes Immunohistochemistry reveals β1-adrenoceptor in splenic tissues for one representative rat per group. The representative sections are shown at×400 original magnification and scale bars are 20 mm.
